# Upgrading Polyurethanes into Functional Ureas through the Asymmetric Chemical Deconstruction of Carbamates

**DOI:** 10.1021/acssuschemeng.2c05647

**Published:** 2022-12-27

**Authors:** Ion Olazabal, Alba González, Saúl Vallejos, Iván Rivilla, Coralie Jehanno, Haritz Sardon

**Affiliations:** † POLYMAT, University of the Basque Country UPV/EHU, Joxe Mari Korta Center, Avda. Tolosa 72, 20018 Donostia-San Sebastian, Spain; ‡ Department of Chemistry, Faculty of Science, University of Burgos, Plaza Misael Bañuelos s/n, 09001 Burgos, Spain; § CQC-IMS, Department of Chemistry, University of Coimbra, Rua Larga, 3004-535 Coimbra, Portugal; ∥ Departamento de Química Orgánica I, Centro de Innovación en Química Avanzada (ORFEO−CINQA), Facultad de Química, Universidad del País Vasco/Euskal Herriko Unibertsitatea (UPV/EHU) and Donostia International Physics Center (DIPC), P° Manuel Lardizabal 3, 20018 San Sebastián-Donostia, Spain; ⊥ Ikerbasque, Basque Fundation for Science, 48009 Bilbao, Spain; # POLYKEY, Joxe Mari Korta Center, Avda. Tolosa 72, 20018 Donostia-San Sebastian, Spain

**Keywords:** Polyurethanes, Recycling, Organocatalysis, Aminolysis, Hindered urea

## Abstract

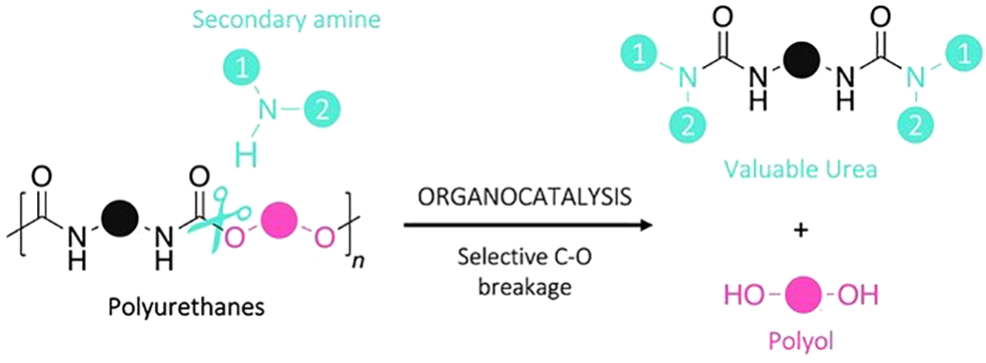

The importance of systematic and efficient recycling of all forms of plastic is no longer a matter for debate. Constituting the sixth most produced polymer family worldwide, polyurethanes, which are used in a broad variety of applications (buildings, electronics, adhesives, sealants, etc.), are particularly important to recycle. In this study, polyurethanes are selectively recycled to obtain high value-added molecules. It is demonstrated that depolymerization reactions performed with secondary amines selectively cleave the C–O bond of the urethane group, while primary amines unselectively break C–O and C–N bonds. The selective cleavage of C–O bonds, catalyzed by an acid:base mixture, led to the initial polyol and a functional diurea in several hours to a few minutes for both model polyurethanes and commercial polyurethane foams. Different secondary amines were employed as nucleophiles to synthesize a small library of diureas obtained in good to excellent yields. This study not only targets the recovery of the initial polyol but also aims to form new diureas which are useful building blocks for the polymerization of innovative materials.

## Abstract 2

This study describes the selective cleavage of C−O bonds in polyurethanes catalyzed by an acid:base mixture leading to the initial polyol and a functional diurea.

## Introduction

Polyurethanes (PUs) constitute one of the most important families of polymers with more than 20 million tons produced in 2019, making them the sixth most produced polymer globally.^1^ This versatile family of materials can be processed as rigid foams, flexible foams, or elastomers, which are important materials for very diverse applications.^2^ Flexible PUs are the materials of choice for insulation panels, tires, and synthetic fibers, while rigid foams are converted into electronic components for consumer goods and the automotive and construction industries. Elastomeric PUs are preferred for coatings, adhesives, surfactants, and elastomers (the so-called CASE applications). Depending on the final material targeted, a PU can be thermoset or thermoplastic, but all types are generally prepared from an isocyanate and a polyol condensation reaction. Considering the enormous scale of PU production, appropriate end-of-life management of these polymers is critical from an environmental point of view. End-of-life options are also important from a a financial viewpoint as the PU industry represents more than US$56 B globally (in 2020) and is projected to reach US$82 B by 2028.^3^ As a result, the improvement of recycling options for PUs is being increasingly called for by leading organizations; the European Isocyanate and Polyol Producers Association (ISOPA), the European association of flexible polyurethane foam blocks manufacturers (EuroPUR), and the Center for the Polyurethane Industry (CPI) are inciting their recycling.^4−6^

The various potential combinations of polyols and isocyanates leads to a myriad of distinctive structures which are key to the use of PUs in such a large range of applications. At the same time, it complicates the recycling process which is affected by physical factors, such as the density of the material or its physical form (e.g., foam, powder, or laminate) as well as the nature of the isocyanate (aromatic or aliphatic) and the nature of the polyol (polyester or polyether). Initial attempts at recycling PUs were based on mechanical recycling as it is the easiest and most straightforward technology to recycle plastics.^7,8^ However, as PUs are mostly produced as thermosets, they cannot be recycled using conventional mechanical methods (e.g., regrinding, powdering, or compression molding), which renders chemical recycling a useful alternative.

**Scheme 1:**
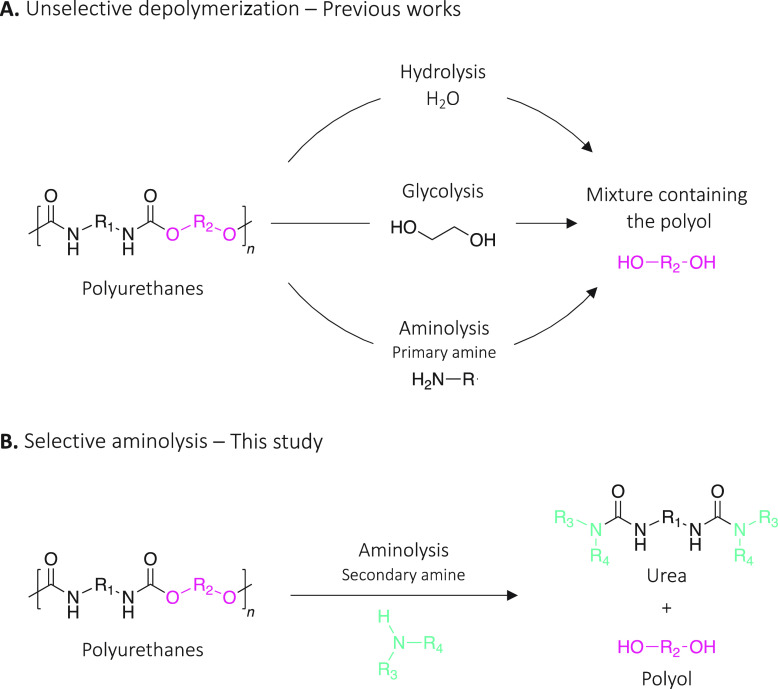
(A) Common Depolymerization Pathways Resulting in a Mixture of Molecules Containing Polyol(s) and (B) Selective Depolymerization Through Aminolysis Proposed in This Study

Despite the numerous examples in the literature describing chemical recycling of commodity polymers,^9−13^ the depolymerization of PUs remains relatively unexplored.^1,14^ Only a limited number of examples are available in the literature which includes hydrolysis,^15^ glycolysis,^16,17^ methanolysis,^18,19^ other types of alcoholysis,^20^ or aminolysis^21^ (Scheme 1A). One of the main issues with solvolysis reactions is that harsh conditions are required for the reaction to be completed in a reasonable amount of time, including high pressures, high temperatures, and/or the use of toxic catalysts. Moreover, most of these depolymerizations consist of unselective cleavages along the PU chain, and even if the depolymerization is successful and the PU is cleaved into smaller pieces, the final product is generally a nonselective mixture from which only the polyol fragments can eventually be valorized.

Although it was the first method to be explored for the depolymerization of flexible PU foams, hydrolysis was rapidly discarded as an option because of the high pressures and temperatures required.^22,23^ The methanolysis process suffers from similar issues as the reactions are typically conducted at temperatures >200 °C, which requires the use of supercritical methanol.^18,19^ Glycolysis has been the most studied process, and various catalysts including alkaline salts^24,25^ and organometallic complexes^26−28^ have been employed. For both types of catalysis, the products obtained are rarely selective, and the amine(s) and polyol(s) formed prevents the possibility of recovering the carbamate function.

More recent initiatives have investigated alternative routes to extract value from PU wastes, such as through hydrogenolysis,^29−31^ acidolysis,^32,33^ or transcarbamoylation.^34^ Hydrogenolysis is limited to the recovery of the polyol and the amine constituents of the PU (the carbonyl fragment is lost during the reaction). Acidolysis allows for the preservation of the urethane group, but the nonselectivity of the reaction leads to side reactions, resulting in a mixture of distinct molecules or oligomers. The transcabarmoylation reaction, which consists of the conversion of a carbamate in another carbamate, is limited to only a few available reagents.

Although it has been largely underexplored, aminolysis is a promising alternative to these strategies for two main reasons, (1) because of the superior nucleophilicity of the amine group which leads to higher reactivity, making it much more suitable for degradation of the polymeric chains; (2) because the low price and high availability of amines means that aminolysis reactions are more easily compatible with industrialization. Aminolysis can be performed with aliphatic amines, ammonia, or alkanolamines (e.g., ethanolamine, diethyl amine, dibutylamine, etc.) at atmospheric pressure and lower temperatures than glycolysis to yield polyamines, carbamates, and polyols.^14^ Pioneering works involving aminoalcohols (ethanolamine and diethanolamine mainly) have suggested that alcoholysis was occurring over aminolysis, the amine only acting as a cocatalyst.^35−37^ Other publications have coupled alcoholysis with aminolysis for obtaining polyols of higher quality from extrusion of the recycled material, using diethanolamine as a “decomposing agent” rather than as a nucleophile.^38,39^ Only a very limited number of examples have been reported where an amine is solely used as nucleophile. For example, diethylenetriamine coupled with sodium hydroxide (NaOH) has been reported as a catalyst for depolymerizing rigid PUs,^40^ and butylamine without catalysts at high temperatures has been used to depolymerize elastomeric PUs.^41^ However, these reactions lead to unselective breaks along the polymer backbone and cause rearrangements along the polymeric chain. As a result again, only the initial polyol can be recovered in these systems.

Herein, a method was developed to chemically deconstruct aromatic and cycloaliphatic PUs in a controlled manner by the selective cleavage of the C–O bond of the urethane function (Scheme 1B). This route not only leads to the recovery of the initial polyol but also generates a diurea, which permits the preservation of the valuable carbonyl and allows for subsequent polymerization into PU-like materials. It should be mentioned that additionally some hindered ureas have shown excellent performance for dynamic polymers.^42^ An acid:base mixture based on an organic base (triazabicyclodecene; TBD) and an organic acid (methanesulfonic acid; MSA), which has already been proven to be efficient for the individual depolymerization of both poly(ethylene terephthalate) (PET)^46^ and Bisphenol-based Polycarbonate (BPA-PC),^47^ is used for the deconstruction of PUs through the nucleophilic attack of secondary amines. The catalyst allows for an increase in the rate of depolymerization without compromising the selectivity toward the final product, thus substantially facilitating the work up after the depolymerization. Finally, this process is applied with success to commercial PU foams, providing another end-of-life option for this important class of commercial polymers.

## Materials and Methods

### Materials

#### Reagents and Solvents

Isophorone diisocyanate (IPDI), toluene diisocyanate (TDI), 1,8-octanediol, hexamethylenediamine, ethylenediamine, 2-(methylamino)ethanol, ethanolamine, isophorone diamine, diethanolamine, *p*-xylene diamine, *N,N,N*-trimethylethane-1,2-diamine, morpholine, 1,5,7-Triazabicycl[4.4.0]dec-5ene (TBD), 1,8-Diazabicyclo[5.4.0]undec-7-ene (DBU), benzoic acid, methanesulfonic acid (MSA), glycerol, and Desmopan were purchased from Sigma-Aldrich or Fisher Scientific. Solvents (technical grade) were purchased from Scharlab. Deuterated DMSO (DMSO-*d*_6_) was purchased from Euroisotop. All materials were used without further purification.

#### Commercial PU Foams

All foams are postindustrial PU waste which was provided by the University of Burgos and the technological center GAIKER. **CPU-F1** is based on TDI and contains carbon black as additive; no further information was provided by the supplier (FTIR spectroscopy was not possible to perform because of the high carbon black content). **CPU-F2** is based on methylene diphenyl diisocyanate (MDI) and was synthesized by the University of Burgos with water and a trifunctional polyol as cross-linker along with a silicon-based surfactant under standard production parameters in the polyurethane industry. No further information was available (Figure S2). **CPU-F3** is based on TDI and a trifunctional polyol; it contains 1.2% of inorganic fillers including 0.4% of titanium dioxide and 0.8% of silicates. The thermal analysis shows a maximum degradation temperature of 349 °C and a *T*_g_ of 78 °C (Figure S3). **CPU-F4** was synthesized from a trifunctional polyol and an aliphatic isocyanate; it contains 9.3% of inorganic fillers including titanium, barium, and tin compounds as well as some silicates. According to the data provided by the supplier, it possesses a maximum degradation temperature of 393 °C and a *T*_g_ of 66 °C (Figure S4).

### Characterization Methods

#### ^1^H and ^13^C Nuclear Magnetic Resonance (NMR)

^1^H NMR spectroscopic measurements were carried out on a Bruker Advance 400 (400 MHz) spectrometer using deuterated DMSO (DMSO-*d*_6_) as solvent at ambient temperature (298 K).

#### Fourier Transformation Infrared Spectra (FT-IR)

FT-IR spectra were obtained by an FT-IR spectrophotometer (Nicolet 6700 FT-IR, Thermo Scientific Inc., USA) using an attenuated total reflectance (ATR) technique (Golden Gate, spectra Tech). Spectra were recorded between 4000 and 525 cm^–1^ with a spectrum resolution of 4 cm^–1^. All spectra were averaged over 10 scans.

#### High-Performance Liquid Chromatography–Mass Spectrometry (HPLC-MS)

Experiments were performed in waters Alliance HPLC-QDA employing a C18 5 μm column with an injection volume of 50 μL using a mixture of 90% water, 10% of acetonitrile, and 0.1% of trifluoroacetic acid running for 60 min (*F* = 0.5 mL). Each compound present in the mixture was analyzed separately to confirm their behavior and characteristic signals on the mass spectra and compared with the crude reaction mixture.

#### Gas Permeation Chromatography (GPC)

GPC analysis (Agilent PL-GPC 50) was performed using a Shodex GPC HFIP-803 (300 × 8.0 mm^2^) with THF as the eluent with a flow rate of 1 mL·min^–1^ with polystyrene standards.

### Synthesis of IPDI-PU

In a typical procedure, 5.00 g of isophorone diisocyanate (IPDI) (22.5 mmol) was introduced in a single neck round-bottom flask. A solution of 1,8-octanediol was prepared by dissolving 3.27 g (22.7 mmol) of 1,8-octanediol in 15 mL of dry THF, and this solution was loaded into an addition funnel. The reaction was carried out under nitrogen atmosphere (to prevent the formation of urea moieties) at 80 °C for 24 h and under magnetic stirring. The solvent was evaporated to obtain a white powder which was dried overnight. ^1^H NMR and FTIR spectroscopy as well as GPC were performed, and the recorded data were used to characterize the product. *M*_w_ = 7250 g·mol^–1^. ^1^H NMR (400 MHz, DMSO-*d*_6_) δ (ppm) 7.05 (S, 1H, NH), 6.90 (s, 1H, NH), 3.90 (t, 4H, O–CH_2_), 3.61 (t, 1H, NH–CH), 2.72 (t, 2H, CH_2_–NH), 1.52–1.25 (m, 18H, aliphatic CH_2_–CH_2_), 1.12, 1.07–0.79 (m, 9H, CH_3_–CH_2_) (Figure S7). FTIR 3320 cm^–1^ urethane NH stretching, 1698 cm^–1^ urethane C=O stretching, 1525 cm^–1^ urethane NH amide II stretching (Figure S8).

### Synthesis of TDI-PU

In a typical procedure, 5.00 g of toluene diisocyanate (TDI) (28.8 mmol) was introduced in a single neck round-bottom flask. A solution of 1,8-octanediol was prepared by dissolving 4.24 g (28.9 mmol) of 1,8-octanediol in 20 mL of dry DMF, and this solution was loaded into an addition funnel. The reaction was carried out under a nitrogen atmosphere (to prevent the formation of urea moieties) at 60 °C for 24 h and under magnetic stirring. The solvent was evaporated to obtain a white powder which was dried overnight. ^1^H NMR and FTIR spectroscopy as well as GPC were performed, and the recorded data were used to characterize the product. *M*_w_ = 7600 g·mol^–1^. ^1^H NMR (400 MHz, DMSO-*d*_6_) δ (ppm) 8.84 (S, 1H, NH), 8.73 (S, 1H, NH), 7.50 (S, 1H, NH), 7.10, 7.05, 7.03 (m, 4H, aromatic CH–CH), 4.04 (t, 4H, 0-CH_2_), 2.11, 2.04 (t, 3H, CH_3_–C), 1.60–1.32 (t, 12H, CH_2_–CH_2_) (Figure S9). FTIR 3295 cm^–1^ urethane NH stretching, 1693 cm^–1^ urethane C=O stretching, 1525 cm^–1^ urethane NH amide II stretching (Figure S10).

### General Procedure for PU Depolymerization Reactions

In a typical experiment, 1.00 g of PU (4.48 mmol, 1 equiv) was degraded using the nucleophile in excess (10 equiv) with a certain amount of catalyst (from 0.15 to 0.45 equiv). A 25 mL round-bottom flask equipped with a magnetic stirrer was used for every reaction. The depolymerizations were carried out under atmospheric pressure and a nitrogen atmosphere at 130, 160, or 190 °C for 7 h. Reagents and catalyst were previously loaded in the glovebox before sealing the flask which was then immersed in an oil bath. At the end of the reaction, an aliquot of the crude product was analyzed by ^1^H NMR spectroscopy for identification of the products and determination of both the depolymerization rate and the different products’ yields.

### Synthesis of Commercial-Like PU Foam

In a typical procedure 0.88 g (9.6 mmol) of glycerol, 15.5 g (4.13 mmol) of desmopan 4042BT trifunctional polyol, and 0.045 g (2.5 mmol) of water were mixed in a beaker along with 0.10 g of Tegostab B8110 as surfactant, 0.15 g (1.34 mmol) of DABCO, and 0.04 g (0.063 mmol) of DBTDL. 4 g (23 mmol) of TDI was then added under stirring until foam formed (Figure S1).

## Results and Discussion

The present study was performed on both aliphatic and aromatic PUs prepared from two of the most widely industrially used isocyanates, isophorone diisocyanate (IPDI) and toluene diisocyanate (TDI), and 1,8-octanediol for obtaining representative PUs, i.e., IPDI-PU and TDI-PU.

### Aminolysis of Model Aliphatic Polyurethane with Hexamethylenediamine

As a model for screening the reaction’s parameters, the depolymerization of IPDI-PU was investigated with hexamethylenediamine as nucleophile. The aliphatic IPDI-PU was selected because of the lower reactivity (and thus, higher reaction times) compared to the aromatic TDI-PU, which allowed us to monitor the reaction with greater ease.^43^ Hexamethylenediamine was chosen because of the easily traceable protons in the ^1^H NMR spectra. All experiments were conducted in bulk in a 100 mL flask equipped with a magnetic stirrer under nitrogen atmosphere. A large excess of nucleophile, i.e., 10 eq., was used, corresponding to the minimum quantity required to immerse the PU. The crude product was analyzed by ^1^H NMR spectroscopy, in DMSO-*d*_6_ for 24 h, to evaluate the depolymerization rate (i.e., the formation of 1,8-octanediol) over time.

**Figure 1:**
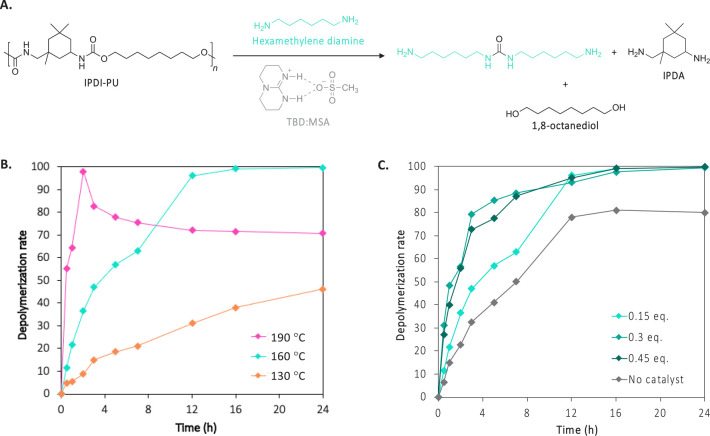
(A) Reaction scheme for the depolymerization of IPDI-PU with hexamethylenediamine. Kinetic plots of the reaction (B) at 130, 160, and 190 °C with 0.15 eq. of TBD:MSA and (C) at 160 °C with 0, 0.15, 0.30, and 0.45 eq. of TBD:MSA. The kinetic was followed by ^1^H NMR spectroscopy in DMSO-*d*_6_ using the characteristic signal of 1,8-octanediol (δ = 3.37 ppm) (Figures S15 to S20). Reaction conditions: IPDI-PU (1 equiv), hexamethylenediamine (10 equiv), N_2_.

Different parameters influencing the depolymerization reaction such as the temperature and the catalyst content were investigated with TBD:MSA as catalyst. TBD:MSA has already been proven to accelerate both polymerization and depolymerization reactions performed at elevated temperatures (with proven thermal stability up to 400 °C), which renders the organic acid:base mixture a suitable candidate for the present study.^46,44,45^ The results shown in Figure 1A suggest that the reaction is highly temperature dependent. At 130 °C, the yield did not exceed 40% after 24 h, while complete depolymerization was afforded in the same time at 160 °C and in 2 h at 190 °C (Figure 1B). However, at this temperature, increased reaction times led to the decrease of the characteristic signals’ intensity of 1,8-octanediol in the ^1^H NMR spectra (δ = 3.37 ppm), which suggests the presence of side reactions (Figure S16). Concomitantly, the intensity of the signal corresponding to the methyl group of isophorone diamine (IPDA) also decreased (δ = 2.15 ppm), which corroborates that undesirable reactions occur between the formed diol and the diamine.

Different loadings of TBD:MSA were also investigated to evaluate the catalytic activity of the organic mixture (Figure 1C). The uncatalyzed depolymerization reached a maximum extent of depolymerization of 80% after 12 h, followed by a plateau up to 24 h. When 0.3 or 0.45 eq. of catalyst were used, the reactions rapidly reached around 85% of conversion (7 h), while the reaction performed with 0.15 eq. of catalyst exhibited lower rates of depolymerization with only 63% conversion at the same reaction time. Ultimately, after 16 h of reaction, the three reactions reached completion without further undesirable side reactions. Therefore, moderate quantities of catalyst are sufficient to efficiently mediate IPDI-PU depolymerizations (Figures S21 and S22).

The results above show that the complete aminolysis of IPDI-PU with hexamethylenediamine can be achieved in less than 24 h assuming use of the appropriate temperature and catalyst. However, using hexamethylenediamine, the depolymerization of IPDI-PU led to three major identified products in the ^1^H NMR spectra, 1,8-octanediol, IPDA, and a linear urea formed through the double nucleophilic attack of the amine on the urethane, 1,3-bis(6-aminohexyl)urea (Figure 1A). The presence of these products suggests that the nucleophilic attack of the urethane carbonyl group breaks both C–N and C–O bonds, demonstrating the nonselectivity of the depolymerization performed with hexamethylenediamine.

### Screening of Amines for the Depolymerization of Polyurethanes

Different amines, i.e., primary and secondary, were screened for the depolymerization of IPDI-PU. The main objective was to avoid the nucleophilic attack on the C–N bond of the urethane and to promote the selective depolymerization through C–O bond breaking. This would lead to a diurea segment which can be homopolymerized to synthesize a new PU-like material with no need for isocyanates. The screening of the nucleophiles was primarily performed on the model aliphatic PU (IPDI-PU) by comparing the depolymerization using different nucleophiles. The reactions were performed at 160 °C with 0.15 eq. of TBD:MSA and 10 eq. of the nucleophile for 7 h under a nitrogen atmosphere (Table 1). The depolymerization reactions were monitored by ^1^H NMR spectroscopy in DMSO-*d*_6_ for 24 h.

**Table 1:**
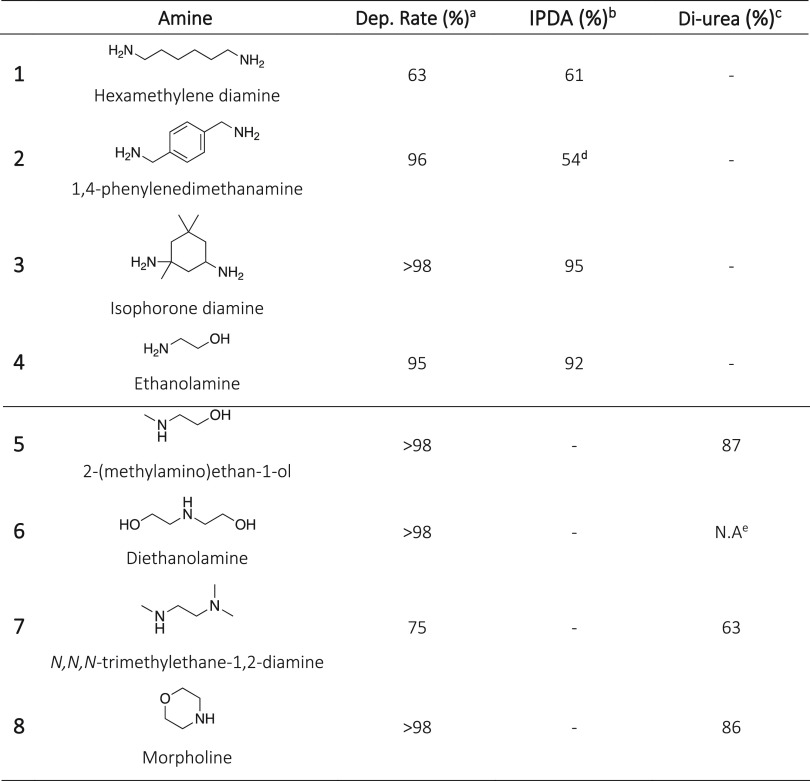
Depolymerization of IPDI-PU Catalyzed by TBD:MSA with Different Amines^f^

a Different ratios were determined by ^1^H NMR spectroscopy in DMSO-*d*_6_ from the crude product using the characteristic signals of 1,8-octanediol (δ = 3.37 ppm).

b Different ratios were determined by ^1^H NMR spectroscopy in DMSO-*d*_6_ from the crude product using the characteristic signals of IPDA (δ = 2.15 ppm).

c Different ratios were determined by ^1^H NMR spectroscopy in DMSO-*d*_6_ from the crude product using the different characteristic signals for the diureas (Figures S24 to S31).

d The poor solubility of the crude product in the deuterated solvent for this reaction could have led to an underestimation of this value.

e The eventual cross-linking of the obtained urea has led to difficulties while determining the yield of the product.

f Reaction conditions: IPDI-PU (1 equiv), amine (10 equiv), catalyst (0.15 equiv) at 160 °C, 7 h, N_2_.

In addition to the use of hexamethylenediamine as described previously, an aromatic, a cyclic aliphatic diamine, and an aminoalcohol were investigated as nucleophiles for the depolymerization of IPDI-PU. All reactions demonstrated similar efficiency with conversion exceeding 95% in less than 7 h. However, similar to the reaction performed with hexamethylenediamine, IPDA and the corresponding linear urea were obtained together with 1,8-octanediol, demonstrating an unselective urethane bond cleavage (Table 1, entries 2 to 5).

After unsuccessful attempts to achieve the desired diurea from the depolymerization with primary amines, secondary amines were investigated as nucleophile. It was hypothesized that the higher steric hindrance of the secondary amines compared to primary amines could play a key role on the selective deconstruction of the carbamate group, allowing for the breaking of the C–O bond while preserving the C–N bond. The aminolysis of IPDI-PU with different secondary amines including 2-(methylamino)ethan-1-ol, diethanolamine, *N*,*N*,*N*-trimethylethane-1,2-diamine, and morpholine was investigated (Table 1, entries 6 to 9). The depolymerization was efficient when employing the aminoalcohol with 87% conversion obtained in 7 h. No characteristic signals of IPDA were observed in the ^1^H NMR spectra along with the expected increase of the singlet at δ = 2.80 ppm corresponding to the −CH_3_ group attached to the tertiary nitrogen of the substituted urea. To further confirm the absence of the urethane group and the presence of urea group, the depolymerization was followed by FTIR spectroscopy (Figures S32 and S33). As expected, while the urethane band centered at 1710 cm^–1^ decreased, a new band attributed to the urea group appeared at 1630 cm^–1^. Finally, the crude product was analyzed by HPLC-MS (Figures S34 to S36). The obtained chromatogram corroborates the presence of the diurea obtained from the selective breakage of the C–O bond, e.g., a signal at *m*/*z* of 373.36 corresponding to the diurea as well as a signal at *m*/*z* of 272.24 corresponding to the monourea obtained from the fragmentation of the molecule. The HPLC-MS spectrum of the pure diurea exhibits the same characteristic signal of the monourea which can be attributed to the reported dynamic character of hindered ureas.^42^ No signal characteristic of IPDA can be observed in the chromatogram, confirming unequivocally its absence and the selectivity of the method.

**Scheme 2:**
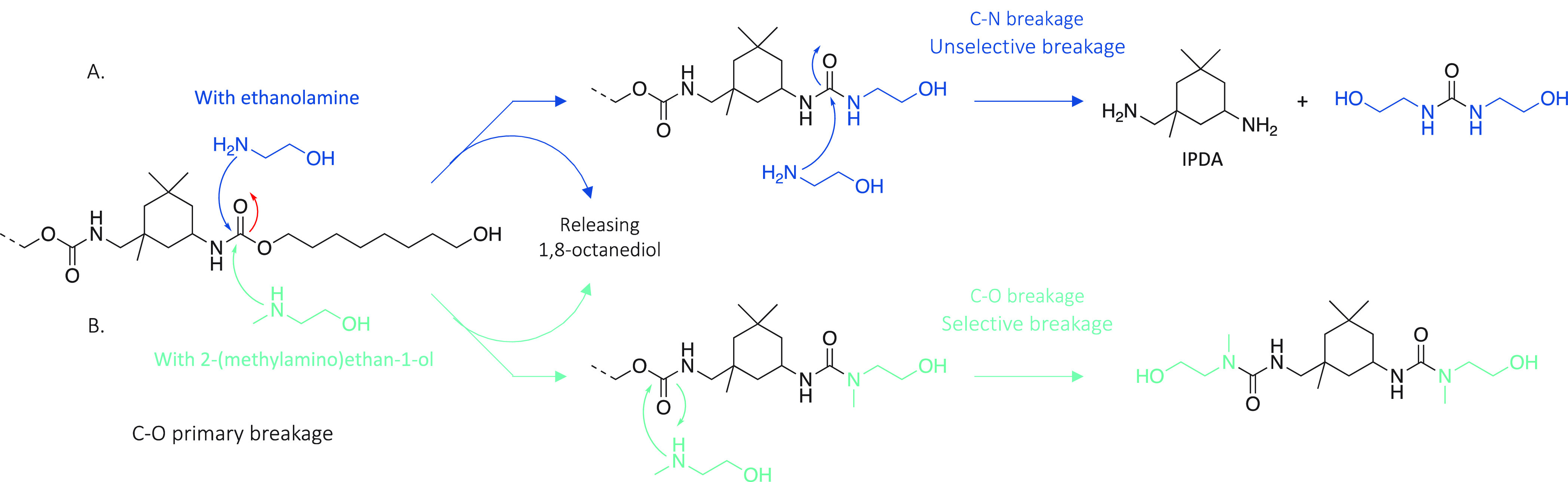
Two Possible Routes for the Depolymerization of IPDI-PU Using Primary or Secondary Amine and the Resulting Products

Similar results were obtained for the other secondary amines. Diethanolamine reacted rapidly and proceeded to full conversion in less than 7 h, but the obtained urea cross-linked at such high temperatures, which rendered the evaluation of the diurea yield impossible. *N,N,N*-Trimethylethane-1,2-diamine only reached 75% of depolymerization conversion while the yield of diurea was about 63% after 7 h. Longer times would have been necessary for completion of the depolymerization. Finally, morpholine presented similar results to 2-(methylamino)ethan-1-ol, reaching complete conversion and 86% of diurea after 7 h. Therefore, while primary amines promote both the C–O and C–N breakage (Scheme 2A), secondary amines exhibit an interesting selectivity that allows degradation of the PU through the unique cleavage of the C–O bond (Scheme 2B). The reaction with 2-(methylamino)ethan-1-ol is of particular interest as it provides a hydroxyl-terminated diurea which can be employed as monomer for further polymerizations.

**Scheme 3:**
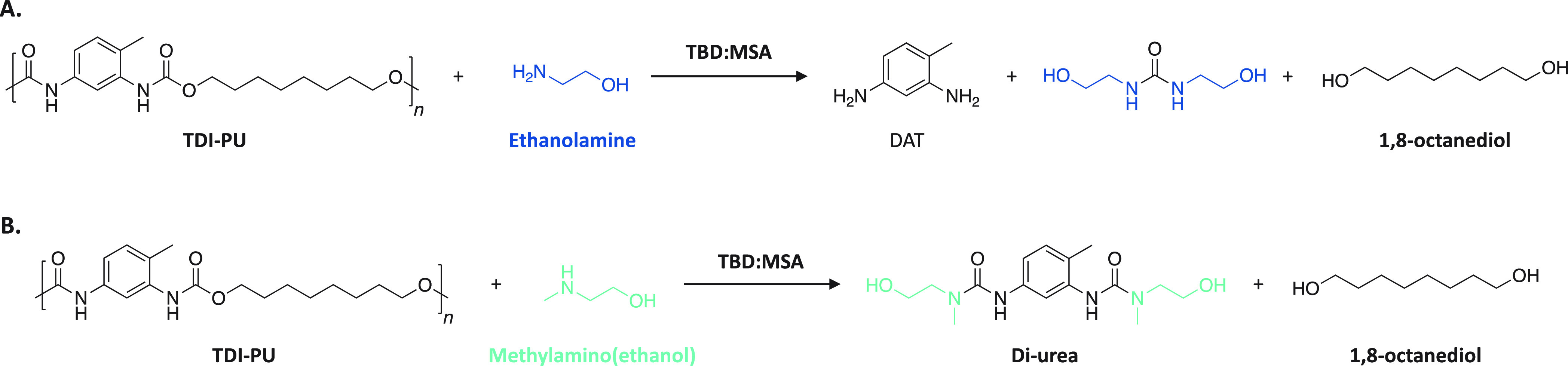
. Reaction Scheme for the Depolymerization of TDI-PU with (A) Ethanolamine and (B) 2-(Methylamino)ethan-1-ol Used As Nucleophile Reaction conditions: TDI-PU (1 equiv), nucleophile (10 equiv), TBD:MSA (0.15 equiv), 40 min, N_2_ (Figures S38 to S40).

To compare the behavior of primary and secondary amines in the depolymerization of aromatic PUs, ethanolamine and 2-(methylamino)ethan-1-ol were used as nucleophiles for the chemical depolymierzation of TDI-PU. Shorter reaction times were observed with completion reached in less than 1 h in both cases. Similar to what was encountered for IPDI-PU, a lack of selectivity was observed when a primary amine was used as nucleophile. For the depolymerization performed with aminoethanol, diaminotoluene (DAT), 1,3-bis(2-hydroxyethyl)urea, and 1,8-octanediol were identified as major products of the reaction in the ^1^H NMR spectra (Scheme 3A). However, when using 2-(methylamino)ethan-1-ol and despite the superior reactivity of aromatic PUs, only 1,8-octanediol and the corresponding diurea were obtained, which confirms that a selective C–O bond cleavage is occurring (Scheme 3B). The extent of depolymerization reached 96% after only 40 min, demonstrating the efficiency of the methodology for selective PU depolymerization. This process is even more critical for aromatic PUs since it prevents the release of DAT, which is considered a cancerogenic chemical.

### Optimization of the Reaction Parameters with (Methylamino)ethan-1-ol

**Scheme 4:**
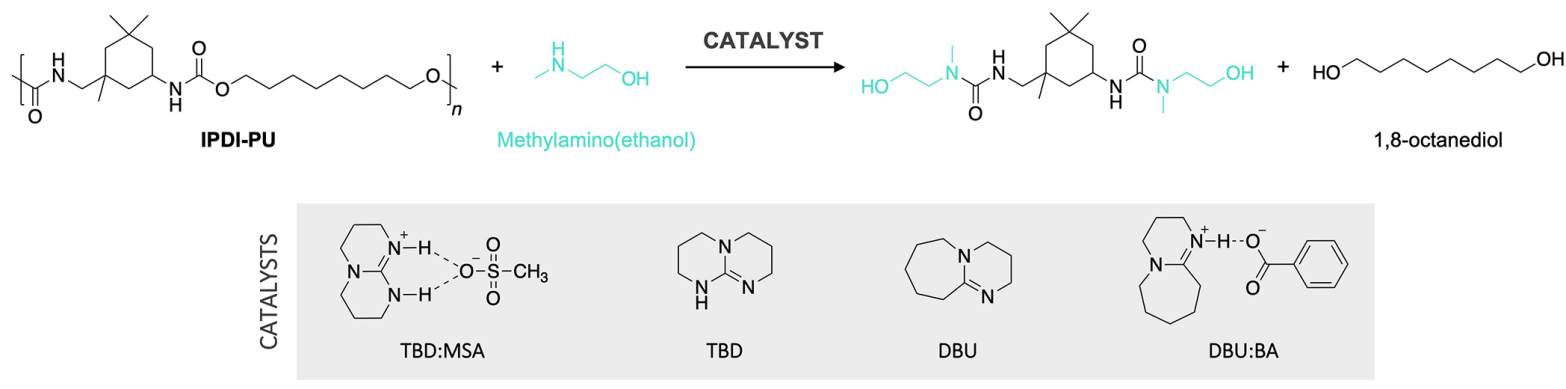
Reaction Scheme for the Depolymerization of IPDI-PU with 2-(Methylamino)ethan-1-ol Catalyzed by a Series of Organocatalysts Reaction conditions: IPDI-PU (1 equiv), 2-(methylamino)ethan-1-ol (10 equiv), catalyst (0.15 equiv), 7 h, N_2_.

Different conditions were investigated to optimize the depolymerization process when a secondary amine is used. Organocatalysts including DBU, TBD, or an equimolar mixture of DBU and benzoic acid (DBU:BA) as well as different temperatures were investigated to determine the impact of these parameters on the selective C–O bond cleavage. All reactions were monitored by ^1^H NMR spectroscopy in DMSO-*d*_6_ for 24 h (Scheme 4).

**Figure 2:**
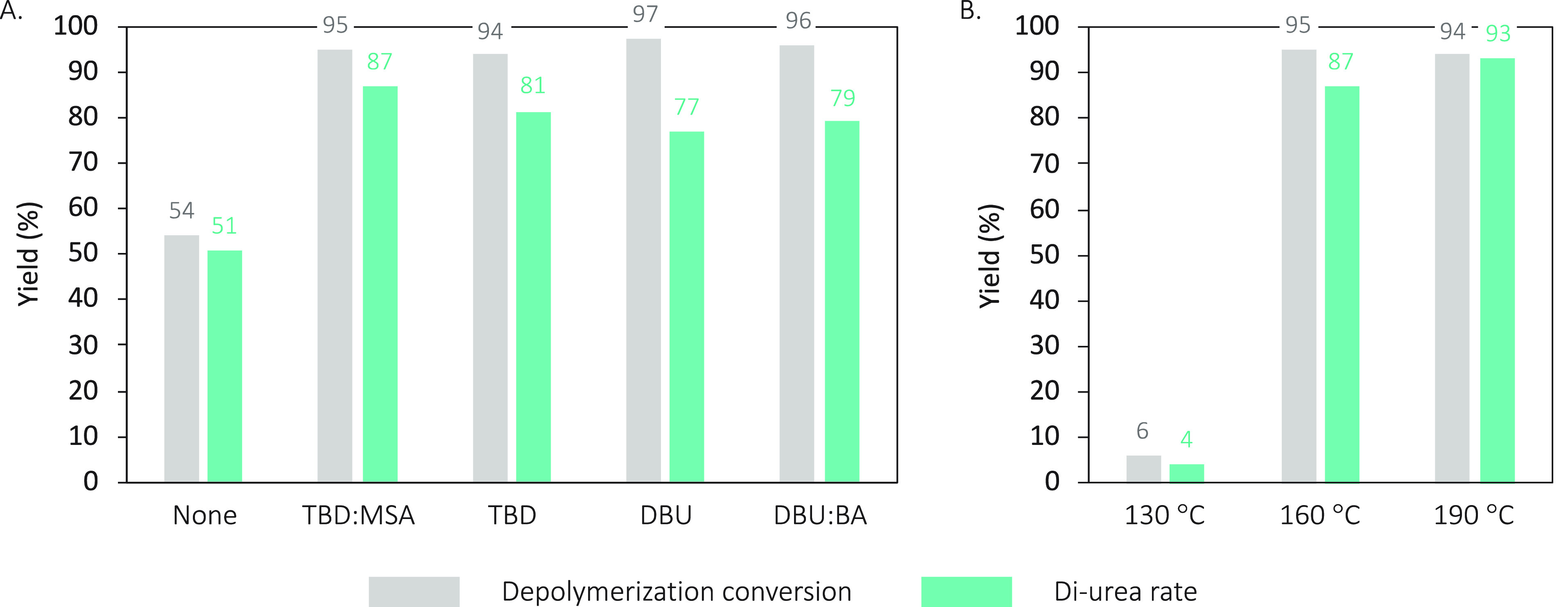
Depolymerization reactions of IPDI-PU with 2-(methylamino)ethan-1-ol catalyzed (A) by a series of organocatalysts at 160 °C and (B) at different temperatures with TBD:MSA. Reaction conditions: IPDI-PU (1 equiv), 2-(methylamino)ethan-1-ol (10 equiv), catalyst (0.15 equiv), 7 h. Ratio determined by ^1^H NMR spectroscopy in DMSO-*d*_6_ from the crude product using the characteristic signals of 1,8-ocatanediol (δ = 3.37) and diurea (δ = 2.81) (Figures S41 to S47).

All catalyzed reactions performed better than the depolymerization without catalyst. The extent of depolymerization was superior to 95% for the catalyzed reactions while the uncatalyzed reaction only reached 54% after 7 h. The performance of the four catalysts was quite similar, but DBU and DBU:BA presented slightly lower conversion into the diurea molecule (77% and 79%, respectively), as compared to TBD and TBD:MSA (81% and 87%, respectively) (Figure 2A). Interestingly, the product of all reactions, including the uncatalyzed depolymerization, was the diurea segment, suggesting that the selective breakage of the PU chain is governed by the nature of the nucleophile employed and not by the catalyst.

The reaction was also performed at different temperatures, i.e., 130, 160, and 190 °C, as it was previously noted that temperature was the most significant parameter (Figure 2B). Surprisingly, the reaction at 130 °C performed extremely poorly. In contrast, reactions at 160 and 190 °C exhibited very similar behaviors, reaching a maximum depolymerization conversions of 95% and 94%, respectively. It can however be noted that the diurea ratio is slightly higher when the reaction is performed at 190 °C, 93%, vs 87% at 160 °C.

### Depolymerization of Commercial Polyurethane Foams

**Figure 3:**
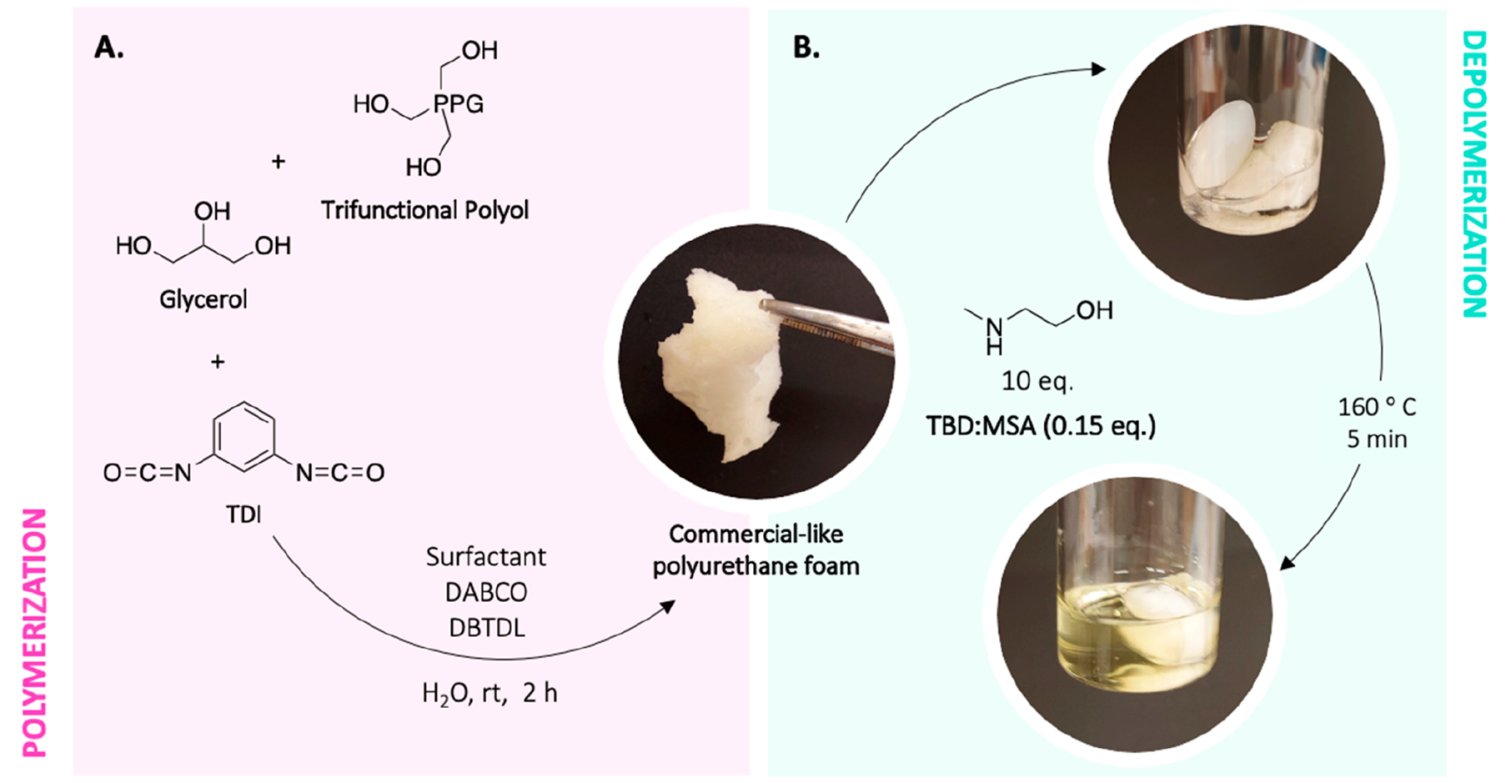
(A) Synthesis scheme of the commercial-like polyurethane foam and (B) its depolymerization employing 2-(methylamino)ethan-1-ol as nucleophile and TBD:MSA as catalyst at 160 °C.

It has been shown that the use of 2-(methylamino)ethan-1-ol as nucleophile and TBD:MSA as catalyst leads to good performances on linear model PUs. To confirm that this observation can be extended to commercially available PUs, the viability of the present depolymerization method was evaluated on a cross-linked PU foam synthesized from TDI, glycerol, and a trifunctional commercial polyol (Figure 3A).

The depolymerization experiment was performed at 160 °C with 0.15 eq. of catalyst for 7 h. The synthesized foam demonstrated complete depolymerization after only 5 min (Figure 3B). The ^1^H NMR spectra of the crude product of this reaction revealed that, also in this case, selective breakage occurred, similar to the examples with the synthesized IPDI-PU and TDI-PU. Interestingly, the depolymerization of the cross-linked TDI-based PU foam seems to be much faster than the corresponding reaction previously performed on TDI-PU (5 min vs 40 min). MALDI-TOF analysis performed on the precipitated polyol obtained from the depolymerization crude product demonstrated no degradation, either in the structure or on the molecular weight (Figure S48). This could be explained by the higher specific surface area of the cross-linked foam material with the reaction media which increases the availability of the urethane groups to react. Regardless of the cross-linked nature of the foam, the reaction was carried out rapidly, demonstrating the effectiveness of the method.

Finally, four different commercial PU foams were investigated for their selective depolymerization with 2-(methylamino)ethan-1-ol under the same conditions mentioned previously (Table 2). **CPU-F1**, which was identified as a cross-linked rigid PU foam based on TDI and an unidentified polyol, did not undergo any depolymerization. After 7 h of reaction, the foam was still intact in the medium with no sign of degradation. On the contrary, the reaction performed on **CPU-F2**, which is a flexible PU foam based on aromatic MDI and a trifunctional polyol, led to the complete depolymerization of the material in 30 min. In the ^1^H NMR spectra, the doublet corresponding to the diurea at δ = 2.76–2.82 ppm can be identified (Figure S49). However, because of the numerous additives and the lack of data on the composition of the foam, a yield cannot be estimated. Here, also, the reaction is even faster than when the model aromatic polyurethane is employed. This again suggests that the higher specific surface area of the flexible foam facilitates depolymerization.

**Table 2:**
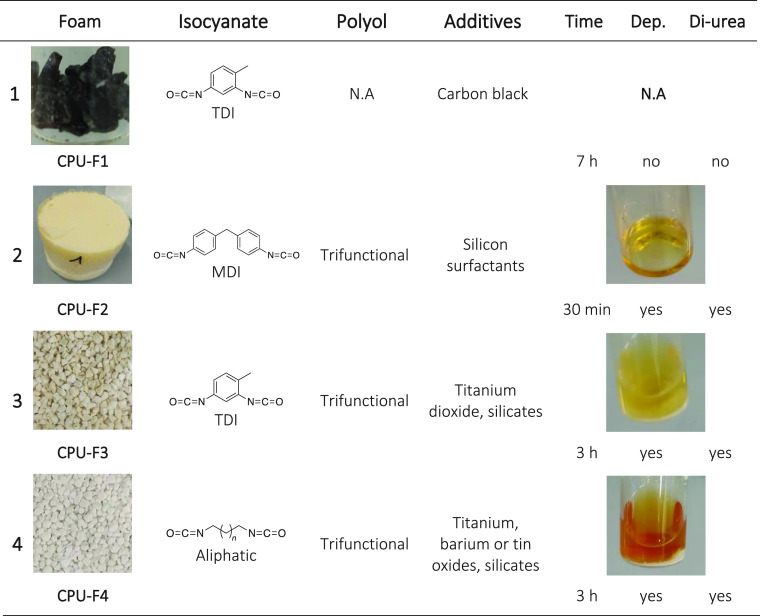
Description of the Different Foams Investigated and Screened Data for Their Depolymerization

**CPU-F3** and **CPU-F4** presented a very similar behavior, regardless of the aliphatic nature of **CPU-F4**. After 3 h, a homogeneous solution containing the insoluble inorganic particles was obtained. The characteristic signal of the diurea can also be observed in the ^1^H NMR spectra of the crude products, highlighting the selectivity of the process (Figures S50 and S51).

These results demonstrate that this procedure can be applied to commercial samples, which makes it a suitable procedure for recycling PU waste. However, it should be highlighted that controlling the additive composition of a material is essential at the recycling stage. Flame retardants, antioxidants, curing agents, UV stabilizers, and much more constitute a long list of chemicals incorporated in small quantities in formulations which are difficult to detect but can disturb the recycling process. In order to efficiently convert discarded PU into a valuable feedstock, more transparency in the composition of the formulations and more eco-design while formulating the materials are necessary.

## Conclusion

In this study, the organocatalytic depolymerization of polyurethanes has been explored with the aim of selectively cleaving the C–O bond of the urethane function. The aminolysis of the polyurethanes, both aliphatic and aromatic, demonstrated high conversion rates with different amines in a process where the nucleophile is employed in excess, under nitrogen atmosphere, and catalyzed by an acid:base catalyst, TBD:MSA. The study has demonstrated that, while primary amines unselectively break the C–O and the C–N bonds, providing an amine and the polyol, secondary amines allow for selective cleavage of the of the C–O moiety to obtain the diurea compound in high yields. This prevents the release of toxic amines and leads to monomers which can be employed for further synthesis of innovative materials. Furthermore, we found that this process could also be implemented for the depolymerization of polyurethane foams, which due to their cross-linked character are impractical for mechanical recycling and are a major contributor to the plastic waste contamination.

## Supporting Information Available

FTIR and ^1^H NMR spectra as well as HPLC-MS and MALDI chromatograms and the complete procedures for model polyurethane synthesis and the different depolymerization reactions (PDF)
