# Supplementary material for: Upgrading Polyurethanes into Functional Ureas through the Asymmetric Chemical Deconstruction of Carbamates
Source: ACS Sustain Chem Eng. 2022 Dec 27;11(1):332–42. doi: 10.1021/acssuschemeng.2c05647 (PMC9832924; doi:10.1021/acssuschemeng.2c05647)
Supplement: Supplementary file 1 — sc2c05647_si_001.pdf [file sc2c05647_si_001.pdf]

Supporting Information

Upgrading polyurethanes into functional ureas through the asymmetric chemical deconstruction of carbamates

Ion Olazabal[1], Alba González[1], Saúl Vallejos,[2,3] Dr. Ivan Rivilla[4,5] Coralie Jehanno[1,6*], Haritz Sardon[1*]

[1]POLYMAT, University of the Basque Country UPV/EHU, Joxe Mari Korta Center, Avda. Tolosa 72, 20018 Donostia-San Sebastian, Spain.

[2] Department of Chemistry, Faculty of Science, University of Burgos, Plaza Misael Bañuelos s/n, 09001 Burgos, Spain

[3] CQC-IMS, Department of Chemistry, University of Coimbra, Rua Larga, 3004-535 Coimbra, Portugal

[4]Departamento de Química Orgánica I, Centro de Innovación en Química Avanzada (ORFEO-CINQA), Facultad de Química, Universidad del País Vasco/Euskal Herriko Unibertsitatea (UPV/EHU) and, Donostia International Physics Center (DIPC), P° Manuel Lardizabal 3, 20018 San Sebastián/Donostia, Spain. [5]Ikerbasque, Basque Fundation for Science, 48009 Bilbao, Spain

[6]POLYKEY, Joxe Mari Korta Center, Avda. Tolosa 72, 20018 Donostia-San Sebastian, Spain.

Number of Pages – 35

Number of Figures – 50

Number of Schemes – 11

Number of tables – 1

FTIR spectra for commercial and commercial-like foams employed in this study

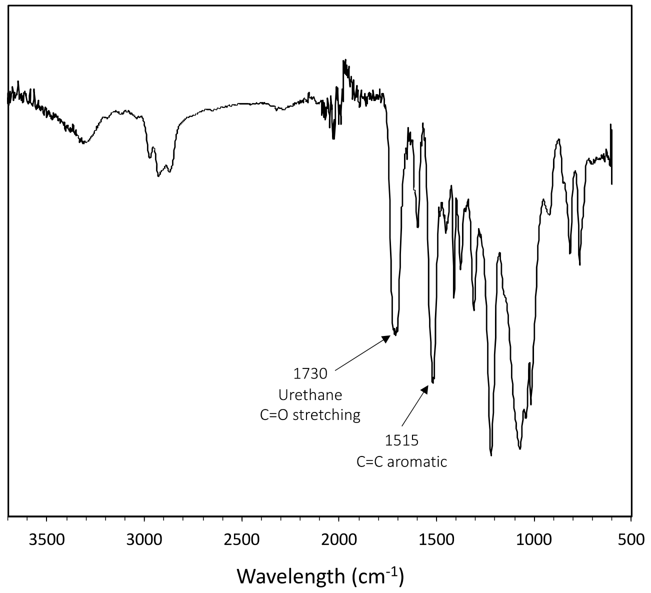

Figure S1. FTIR spectra of the commercial-like foam.

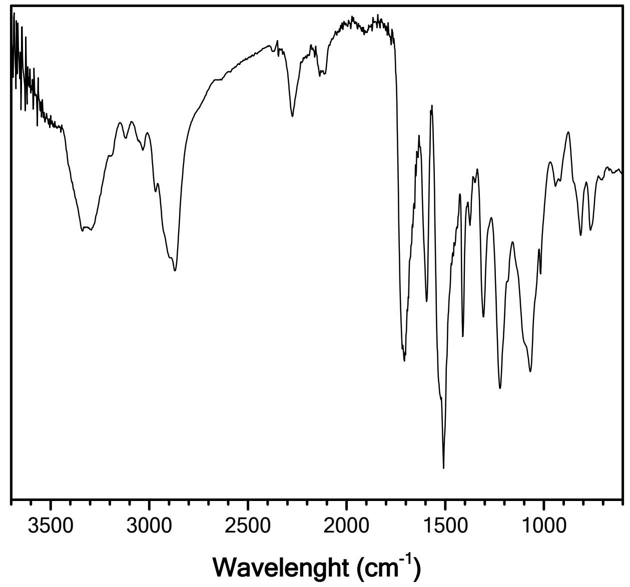

Figure S2. FTIR spectrum of CPU-F2

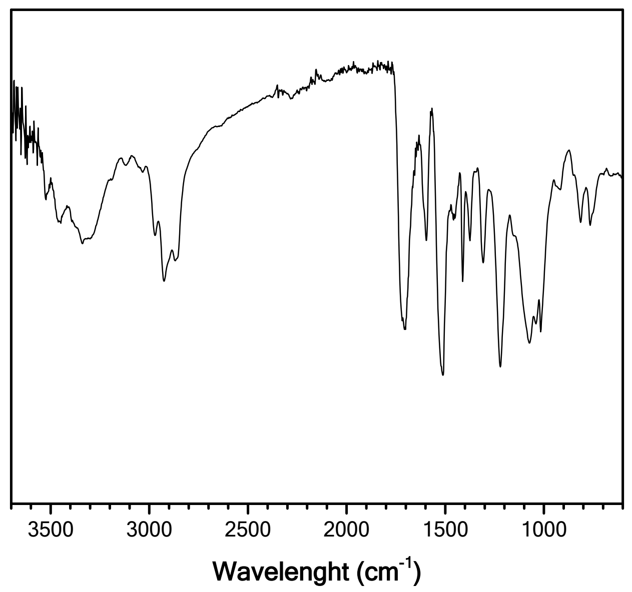

Figure S3. FTIR spectrum of CPU-F3

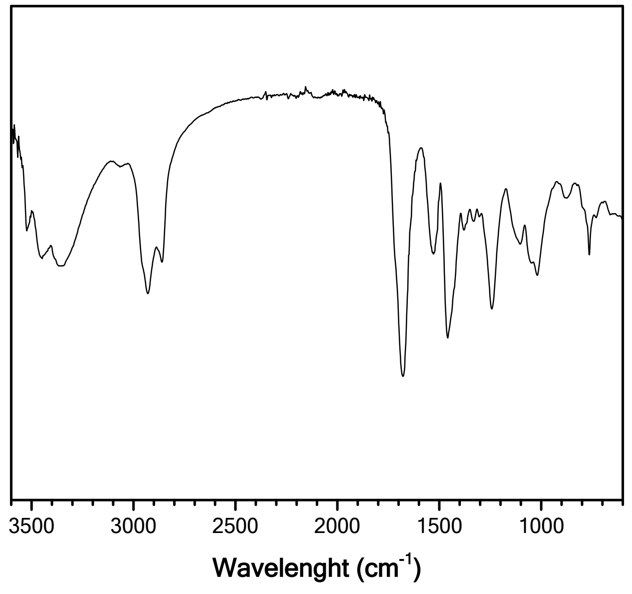

Figure S4. FTIR spectrum of CPU-F4

Catalyst synthesis and characterization

TBD:MSA

In a flame dried rout bottom flask an equimolar quantity of TBD (2.00 g, 14.3 mmol) and MSA (1.38 g, 14.3 mmol) were added and dissolved completely in dry acetone (100mL). The mixture was heated until it became completely transparent. Later the solution kept at room temperature for 24 h to promote the crystallization subsequently it was cooled to 6 °C in a fridge for 24 h yielding transparent crystals whose characterization by ^{1}H NMR and ^{13}C NMR were in good agreement with data previously reported.[1]

^{1}H NMR (400 MHz, DMSO, 298K) δ(ppm) 7.73 (s, 2H, N-H-O), 3.27 (t, 4H, CH_2), 3.16 (t, 4H, CH_2), 2.39 (s, 3H, CH_3), 1.87 (q, 4H, CH_2).

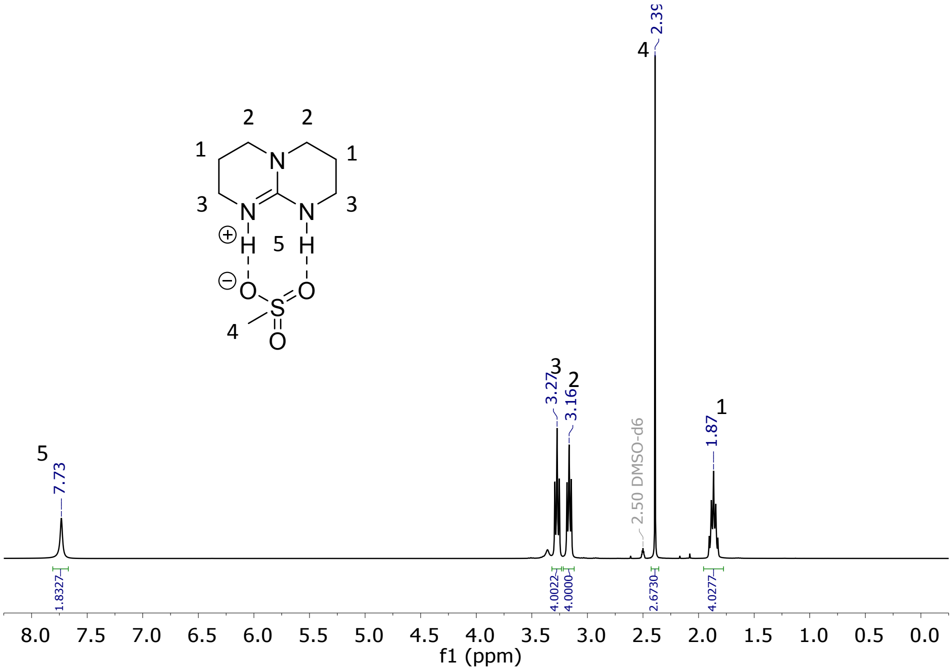

Figure S5. ^{1}H NMR spectrum of TBD:MSA in DMSO-d_6.

DBU:BA

In a flame dried rout bottom flask an equimolar quantity of DBU (2 g, 13.1 mmol) and benzoic acid (1.60 g, 13.1 mmol) were added and dissolved completely in methanol (15 mL). The compound was precipitated by adding an excess of diethyl ether. The powder was filtrated collected and dried prior of being characterized by ^{1}H-NMR were in good agreement with data previously reported.[1]

^{1}H NMR (400 MHz, DMSO, 298K) δ(ppm) 7.83 (s, 2H, CH), 7.28 (s, 3H, CH), 3.53 (dd, 4H, CH_2), 3.28 (s, 2H, CH_2), 2.79 (s, 2H, CH_2), 1.90 (s, 2H, CH_2), 1.63 (q, 4H, CH_2).

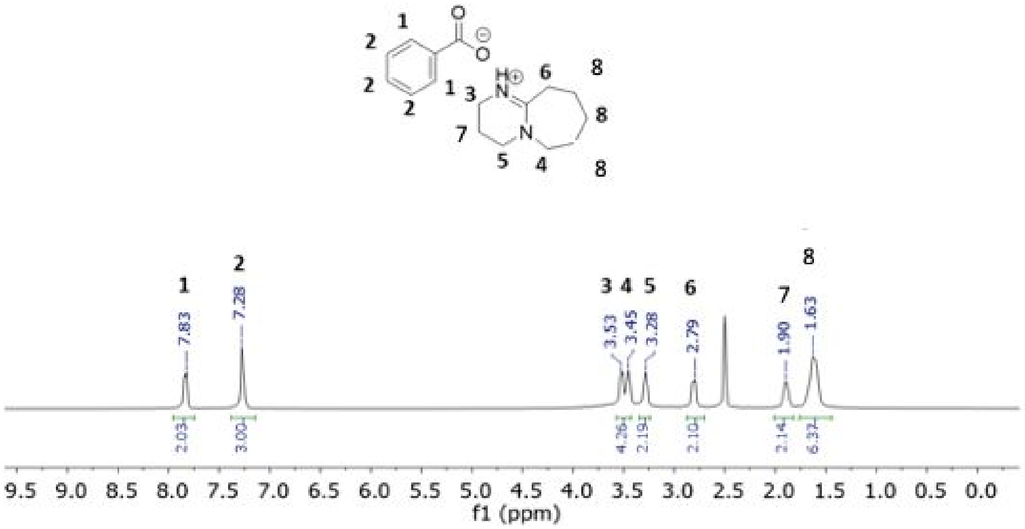

Figure S6. ^{1}H NMR spectrum of DBU:BA in DMSO-d_6.

Model Polyurethanes characterization

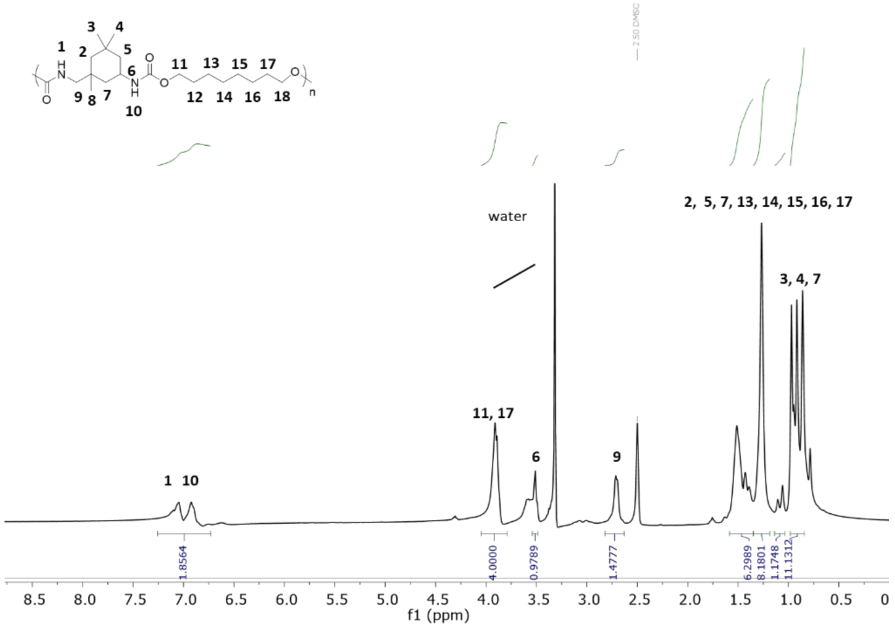

Figure S7. ^{1}H NMR spectrum of IPDI-PU in DMSO-d_6 (300 MHz, 298 K). δ 7.26 – 6.73 (m, 2H), 3.93 (s, 1H), 3.90 (d, J = 6.5 Hz, 2H), 3.50 (d, J = 5.8 Hz, 1H), 2.71 (d, J = 6.4 Hz, 1H), 1.47 (dt, J = 31.1, 9.8 Hz, 5H), 1.27 (s, 7H), 1.08 (d, J = 13.8 Hz, 1H), 1.02 – 0.75 (m, 12H).

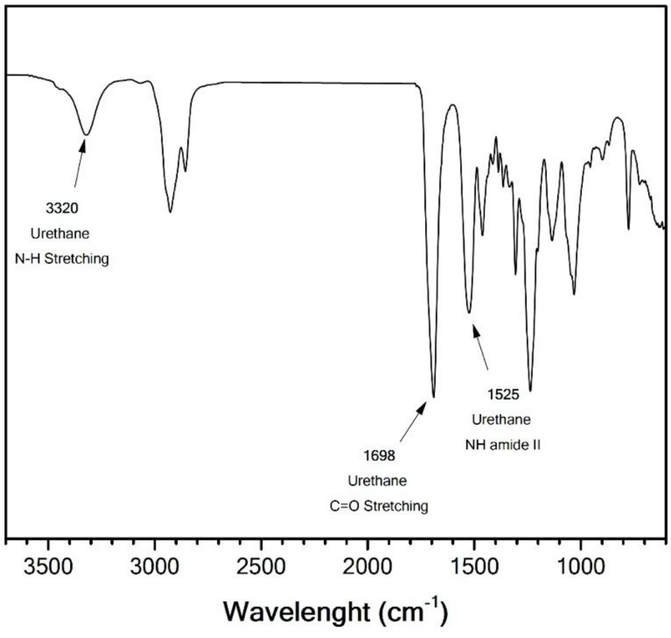

Figure S8. FTIR spectrum of IPDI-PU

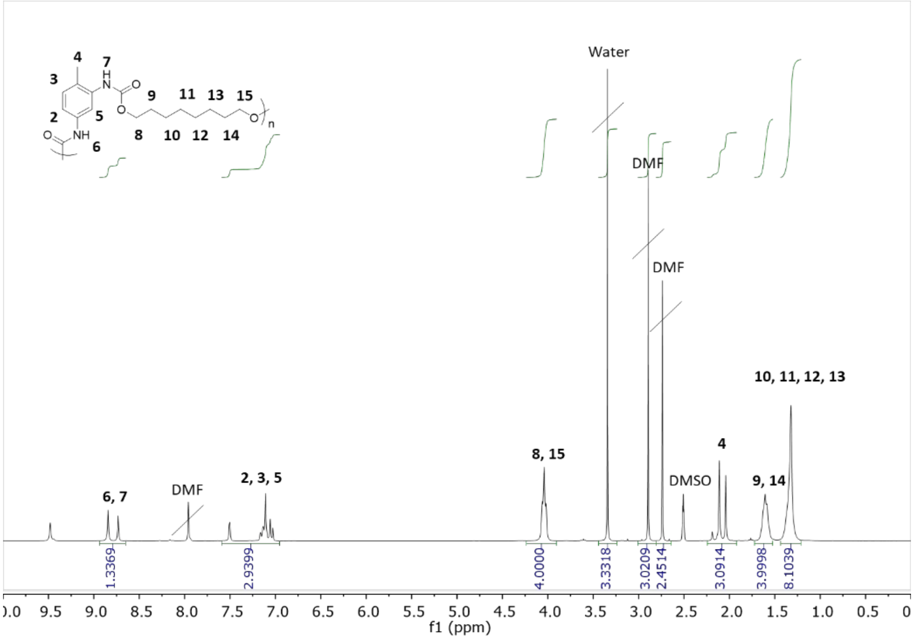

Figure S9. ^{1}H NMR spectrum of TDI-PU in DMSO-d_6 (300 MHz, 298 K). δ 9.58 – 8.61 (m, 2H), 7.69 – 6.89 (m, 3H), 4.05 (q, *J* = 6.6, 4.6 Hz, 4H), 2.08 (d, *J* = 21.3 Hz, 3H), 1.61 (t, *J* = 6.9 Hz, 4H), 1.32 (s, 8H).

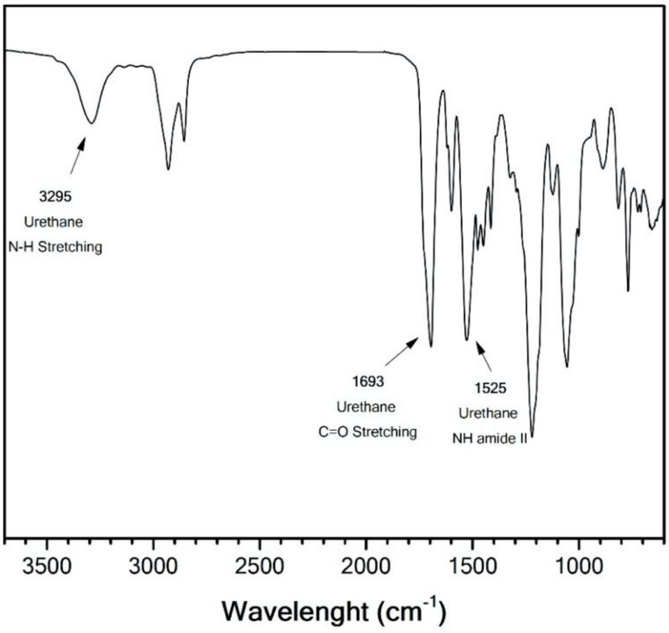

Figure S10. FTIR spectrum of TDI-PU

PU depolymerization reactions

Depolymerization of IPDI-PU with ethylene glycol

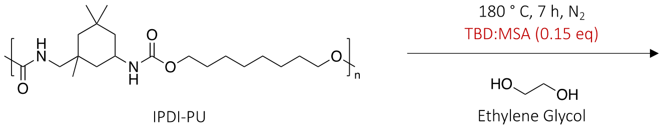

Scheme S1. Depolymerization of IPDI-PU with ethylene glycol at 180 °C with 0.15 eq. of TBD:MSA

1.00 g of PU (4.48 mmol, 1 eq.) are degraded with ethylene glycol (44.8 mmol, 10 eq.) with TBD:MSA (0.672 mmol, 0.15 eq.) in a 25 mL round bottom flask equipped with a magnetic stirrer. The depolymerization is carried out under atmospheric pressure and nitrogen atmosphere at 180 °C for 7 h.

Depolymerization of IPDI-PU with hexamethylene diamine

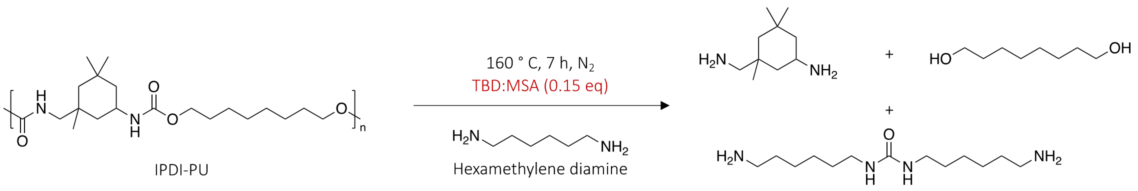

Scheme S2. Depolymerization of IPDI-PU with hexamethylene diamine at 160 °C with 0.15 eq. of TBD:MSA

1.00 g of PU (4.48 mmol, 1 eq.) are degraded with hexamethylene diamine (44.8 mmol, 10 eq.) with TBD:MSA (0.672 mmol, 0.15 eq.) in a 25 mL round bottom flask equipped with a magnetic stirrer. The depolymerization is carried out under atmospheric pressure and nitrogen atmosphere at 160 °C for 7 h.

Depolymerization of IPDI-PU with 1,4-phenylenedimethaneamine

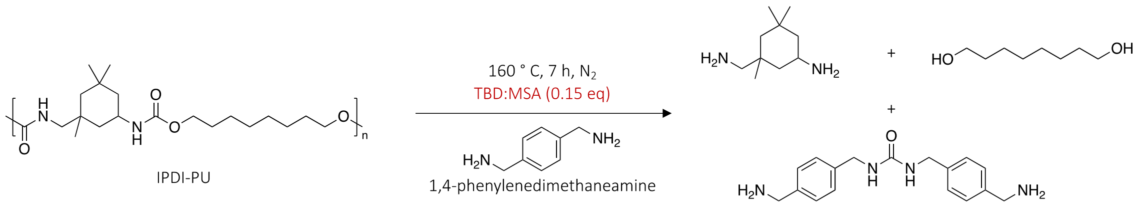

Scheme S3. Depolymerization of IPDI-PU with 1,4-phenylenedimethaneamine at 160 °C with 0.15 eq. of TBD:MSA

1.00 g of PU (4.48 mmol, 1 eq.) are degraded with 1,4-phenylenedimethaneamine (44.8mmol, 10 eq.) with TBD:MSA (0.672 mmol, 0.15 eq.) in a 25 mL round bottom flask equipped with a magnetic stirrer. The depolymerization is carried out under atmospheric pressure and nitrogen atmosphere at 160 °C for 7 h.

Depolymerization of IPDI-PU with isophorone diamine

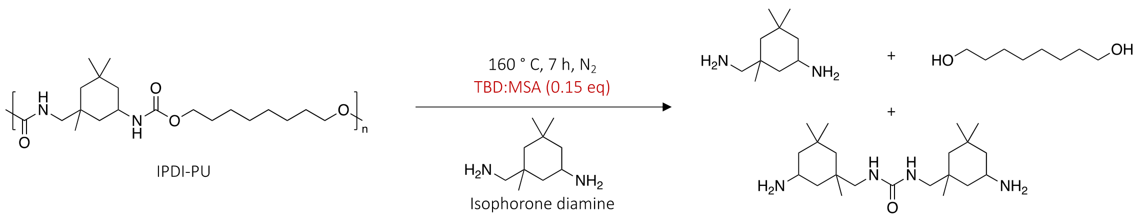

Scheme S4. Depolymerization of IPDI-PU with isophorone diamine at 160 °C with 0.15 eq. of TBD:MSA

1.00 g of PU (4.48 mmol, 1 eq.) are degraded with isophorone diamine (44.8 mmol, 10 eq.) with TBD:MSA (0.672mmol, 0.15 eq.) in a 25 mL round bottom flask equipped with a magnetic stirrer. The depolymerization is carried out under atmospheric pressure and nitrogen atmosphere at 160 °C for 7 h.

Depolymerization of IPDI-PU with ethanolamine

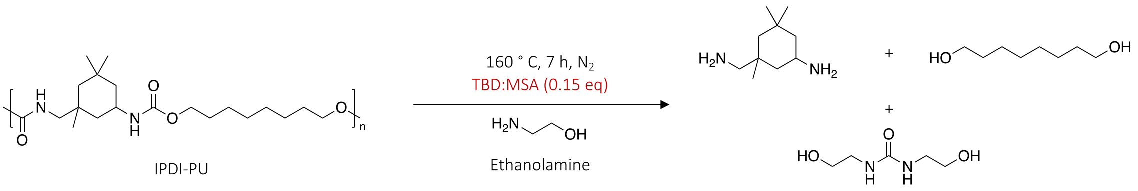

Scheme S5. Depolymerization of IPDI-PU with ethanolamine at 160 °C with 0.15 eq. of TBD:MSA

1.00 g of PU (4.48 mmol, 1 eq.) are degraded with ethanolamine (44.8 mmol, 10 eq.) with TBD:MSA (0.672 mmol, 0.15 eq.) in a 25 mL round bottom flask equipped with a magnetic stirrer. The depolymerization is carried out under atmospheric pressure and nitrogen atmosphere at 160 °C for 7 h.

Depolymerization of IPDI-PU with 2-(methylamino) ethan-1-ol

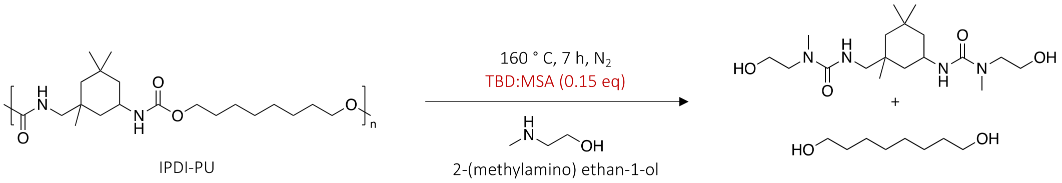

Scheme S6. Depolymerization of IPDI-PU with ethanolamine at 160 °C with 0.15 eq. of TBD:MSA

1.00 g of PU (4.48 mmol, 1 eq.) are degraded with 2-(methylamino) ethan-1-ol (44.8mmol, 10 eq.) with TBD:MSA (0.672 mmol, 0.15 eq.) in a 25 mL round bottom flask equipped with a magnetic stirrer. The depolymerization is carried out under atmospheric pressure and nitrogen atmosphere at 160 °C for 7 h.

Depolymerization of IPDI-PU with diethanolamine

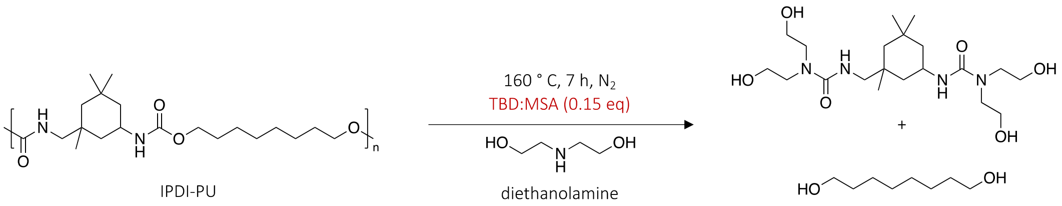

Scheme S7. Depolymerization of IPDI-PU with diethanolamine at 160 °C with 0.15 eq. of TBD:MSA

1.00 g of PU (4.48 mmol, 1 eq.) are degraded with diethanolamine (44.8mmol, 10 eq.) with TBD:MSA (0.672 mmol, 0.15 eq.) in a 25 mL round bottom flask equipped with a magnetic stirrer. The depolymerization is carried out under atmospheric pressure and nitrogen atmosphere at 160 °C for 7 h.

Depolymerization of IPDI-PU with N,N,N-trimethylethane-1,2-diamine

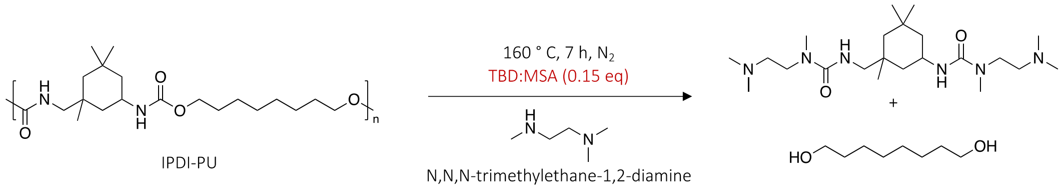

Scheme S8. Depolymerization of IPDI-PU with N,N,N-trimethylethane-1,2-diamine at 160 °C with 0.15 eq. of TBD:MSA

1.00 g of PU (4.48 mmol, 1 eq.) are degraded with N,N,N-trimethylethane-1,2-diamine (44.8 mmol, 10 eq.) with TBD:MSA (0.672 mmol, 0.15 eq.) in a 25 mL round bottom flask equipped with a magnetic stirrer. The depolymerization is carried out under atmospheric pressure and nitrogen atmosphere at 160 °C for 7 h.

Depolymerization of IPDI-PU with morpholine

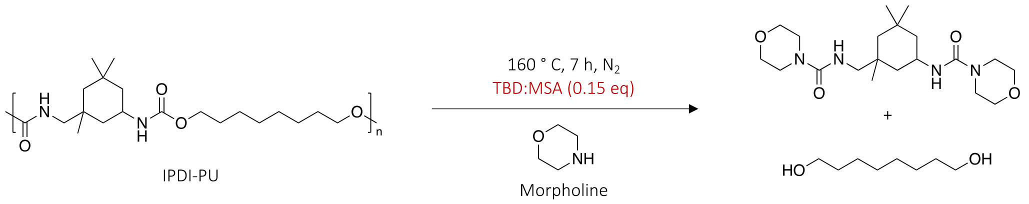

Scheme S9. Depolymerization of IPDI-PU with morpholine at 160 °C with 0.15 eq. of TBD:MSA

1.00 g of PU (4.48 mmol, 1 eq.) are degraded with morpholine (44.8 mmol, 10 eq.) with TBD:MSA (0.672 mmol, 0.15 eq.) in a 25 mL round bottom flask

equipped with a magnetic stirrer. The depolymerization is carried out under atmospheric pressure and nitrogen atmosphere at 160 °C for 7 h.

Depolymerization of TDI-PU with ethanolamine

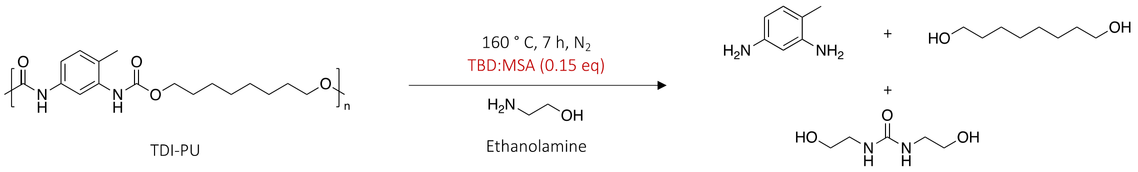

Scheme S10. Depolymerization of TDI-PU with ethanolamine at 160 °C with 0.15 eq. of TBD:MSA

1.00 g of PU (3.13 mmol, 1 eq.) are degraded with ethanolamine (31.3 mmol, 10 eq.) with TBD:MSA (0.468 mmol, 0.15 eq.) in a 25 mL round bottom flask equipped with a magnetic stirrer. The depolymerization is carried out under atmospheric pressure and nitrogen atmosphere at 160 °C for 7 h.

Depolymerization of IPDI-PU with 2-(methylamino) ethan-1-ol

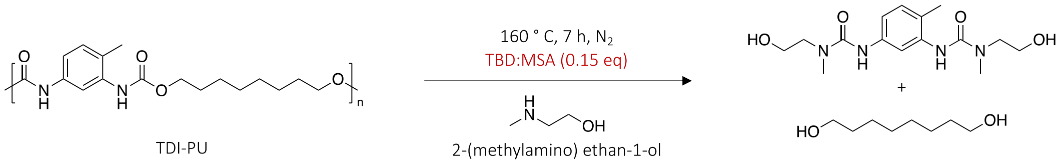

Scheme S11. Depolymerization of TDI-PU with ethanolamine at 160 °C with 0.15 eq. of TBD:MSA

1.00 g of PU (3.13 mmol, 1 eq.) are degraded with 2-(methylamino) ethan-1-ol (31.3 mmol, 10 eq.) with TBD:MSA (0.468 mmol, 0.15 eq.) in a 25 mL round bottom flask equipped with a magnetic stirrer. The depolymerization is carried out under atmospheric pressure and nitrogen atmosphere at 160 °C for 7 h.

Products characterization by means of ^{1}H-NMR

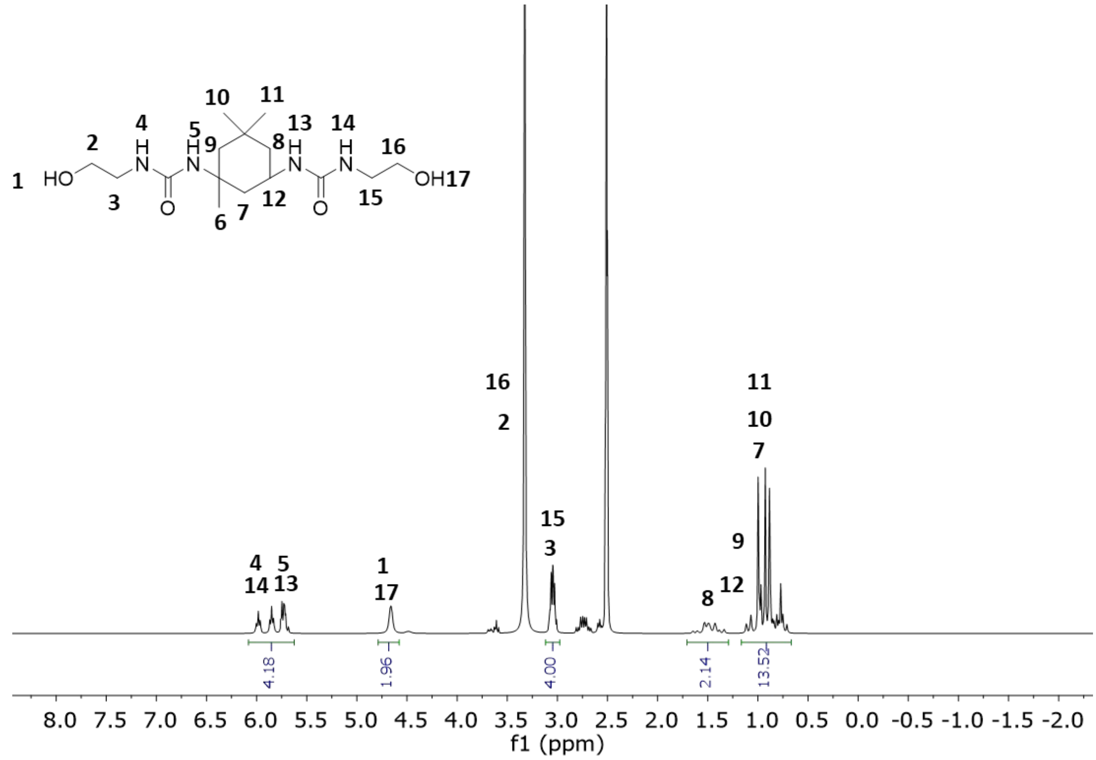

Figure S11. ^{1}H-NMR spectrum for the di-urea resulting from the depolymerization of IPDI-PU with ethanolamine as nucleophile. (300 MHz, DMSO-d_6) δ 6.07 – 5.64 (m, 4H), 4.66 (s, 2H), 3.05 (td, J = 5.9, 4.5 Hz, 4H), 1.65 – 1.31 (m, 2H), 1.16 – 0.66 (m, 13H).

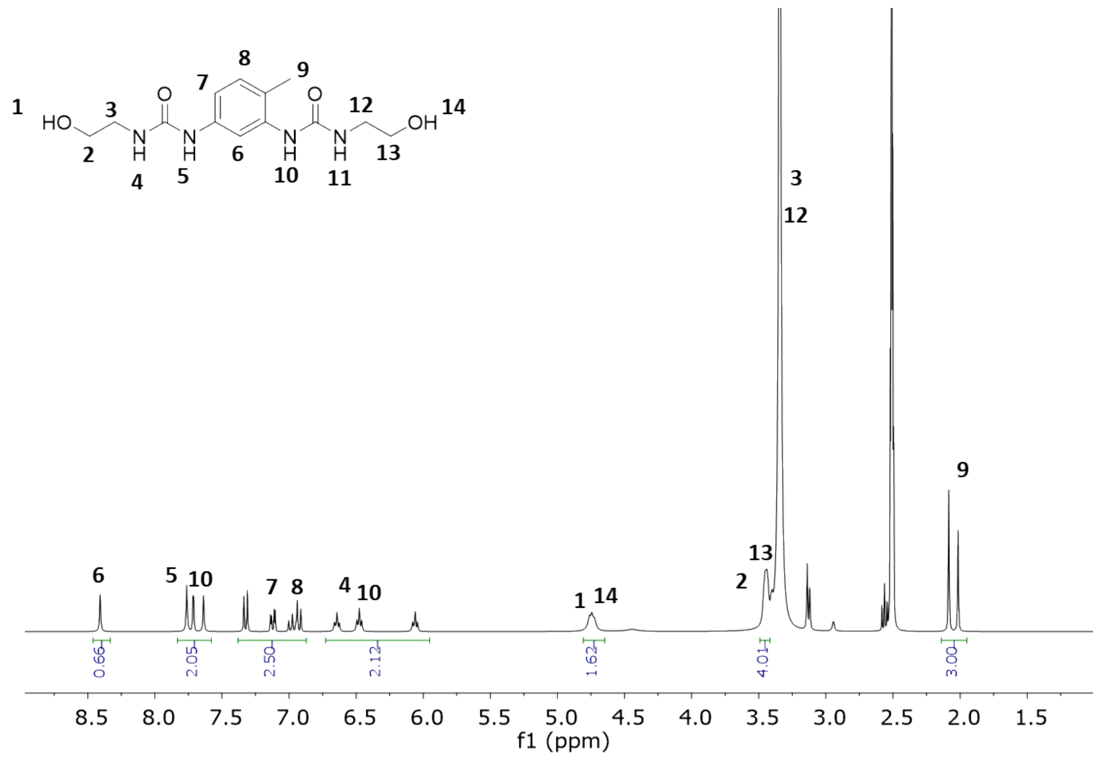

Figure S12. ^{1}H-NMR spectrum for the di-urea resulting from the depolymerization of TDI-PU with ethanolamine as nucleophile. (300 MHz, DMSO-d_6) δ 7.85 – 7.54 (m, 2H), 7.39 – 6.87 (m, 2H), 6.73 – 5.90 (m, 1H), 4.74 (t, J = 6.2 Hz, 1H), 3.52 – 3.42 (m, 4H), 2.05 (d, J = 20.7 Hz, 3H).

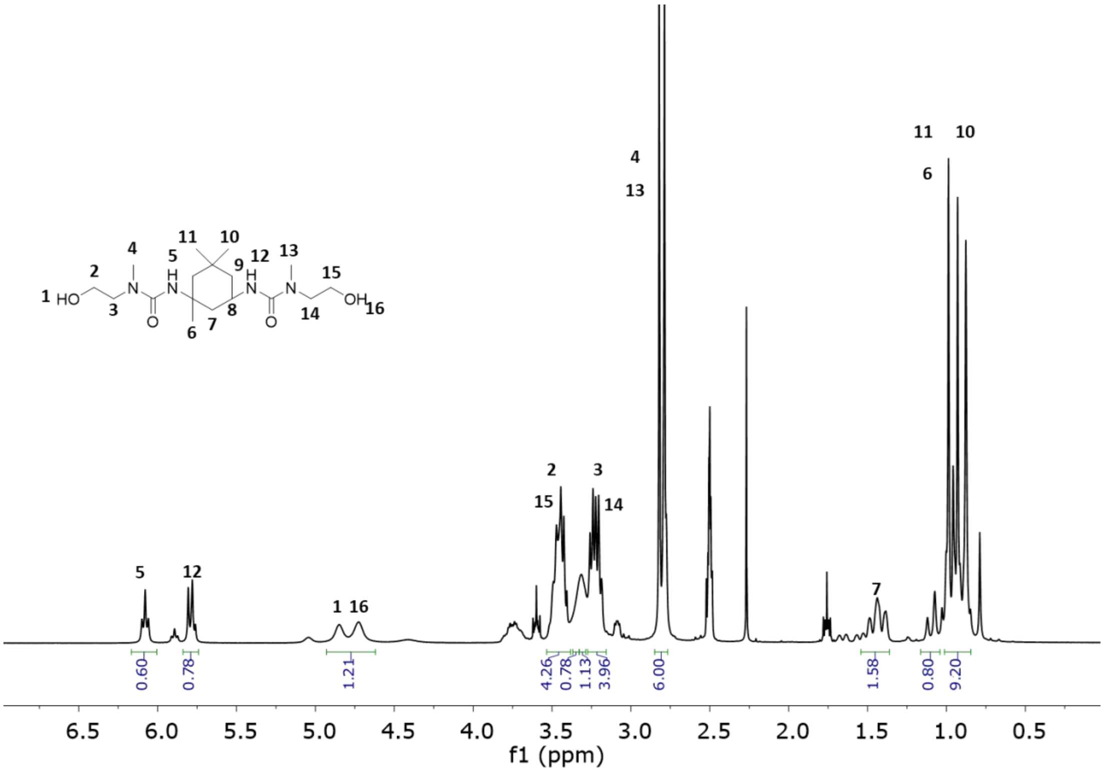

Figure S13. ^{1}H-NMR spectrum for the di-urea resulting from the depolymerization of IPDI-PU with 2-(methylamino)ethan-1-ol as nucleophile. (300 MHz, DMSO-d_6): δ 6.08 (t, J = 6.1 Hz, 1H), 5.79 (d, J = 7.6 Hz, 1H), 4.84 (t, J = 4.9 Hz, 1H), 4.72 (q, J = 4.9 Hz, 1H), 3.75 (d, J = 7.8 Hz, 1H), 3.46 (dd, J = 9.2, 5.0

Hz, 4H), 3.22 (dt, J = 11.4, 5.7 Hz, 4H), 2.89 – 2.72 (m, 6H), 1.44 (t, J = 15.0 Hz, 2H), 1.18 – 0.71 (m, 10H).

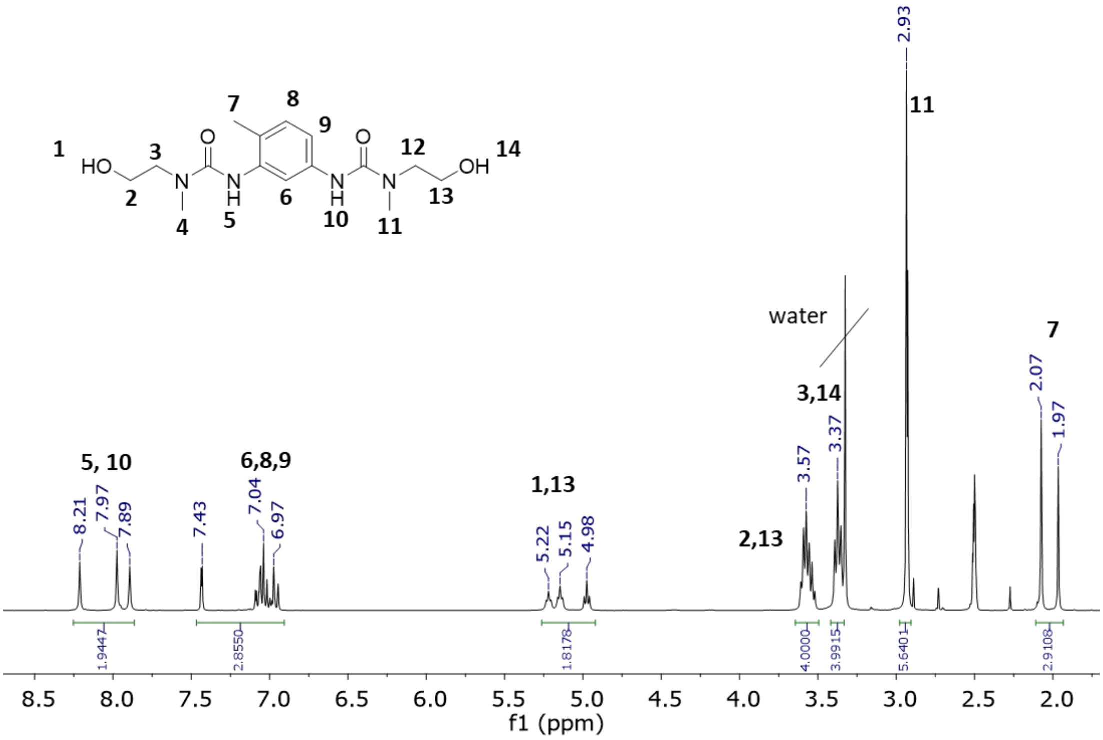

Figure S14. ^{1}H-NMR spectrum for the di-urea resulting from the depolymerization of TDI-PU with 2-(methylamino)ethan-1-ol as nucleophile.(300 MHz, DMSO-d_6): δ 8.25 – 7.86 (m, 2H), 7.47 – 6.91 (m, 3H), 5.26 – 4.92 (m, 2H), 3.57 (s, 4H), 3.37 (s, 4H), 2.93 (s, 6H), 2.02 (d, J = 32.8 Hz, 3H).

Kinetics followed by ^{1}H NMR spectroscopy

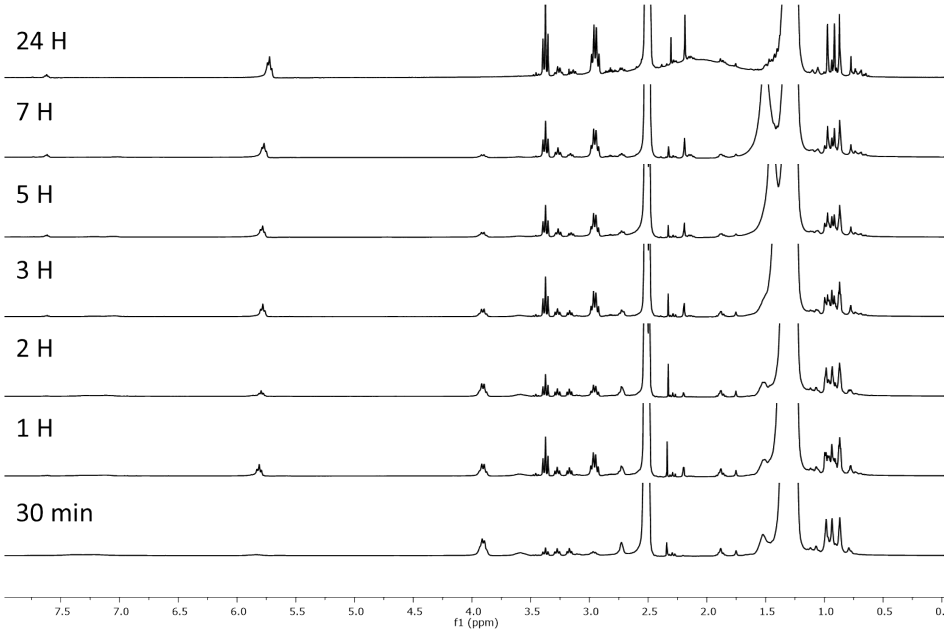

Figure S15. Stacked ^{1}H NMR spectra for the kinetics of IPDI-PU depolymerization with hexamethylenediamine as nucleophile with TBD:MSA as catalyst at 160 °C. (DMSO-d_6, 300 MHz, 298 K)

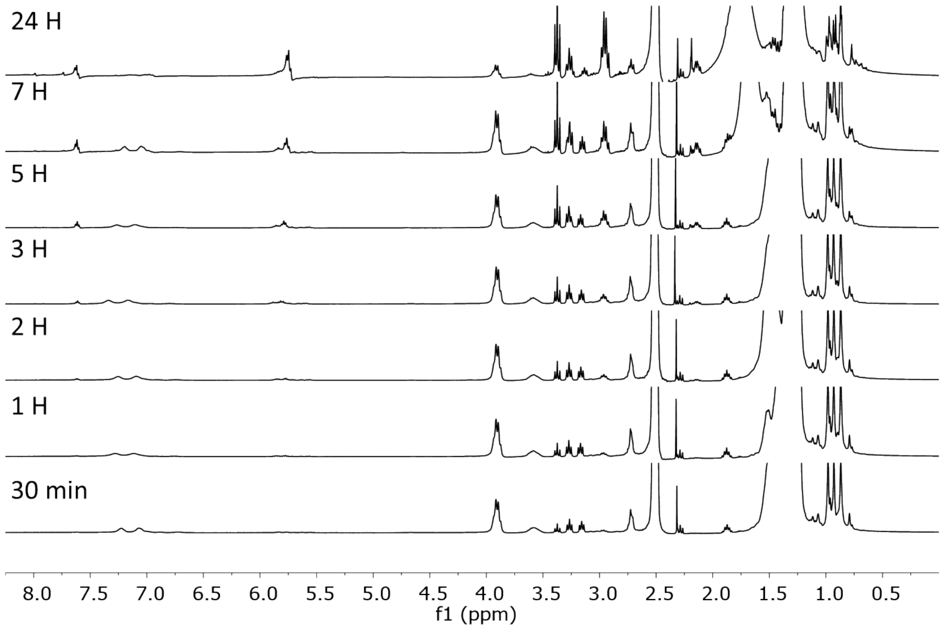

Figure S16. Stacked ^{1}H NMR spectra for the kinetics of IPDI-PU depolymerization with hexamethylenediamine as nucleophile with TBD:MSA as catalyst at 130 °C. (DMSO-d_6, 300 MHz, 298 K)

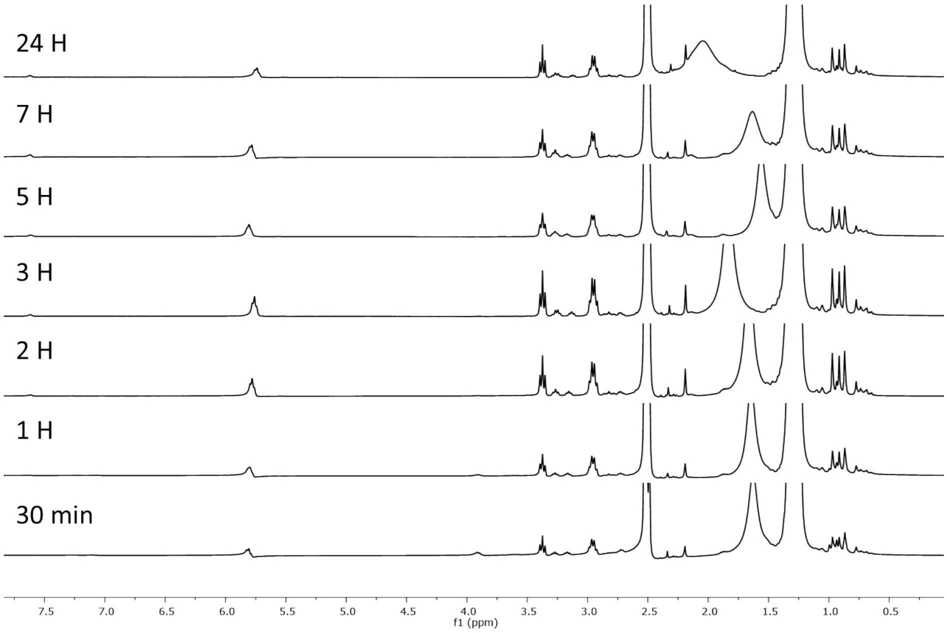

Figure S17. Stacked ^{1}H NMR spectra for the kinetics of IPDI-PU depolymerization with hexamethylenediamine as nucleophile with TBD:MSA as catalyst at 190 °C. (DMSO-d_6, 300 MHz, 298 K)

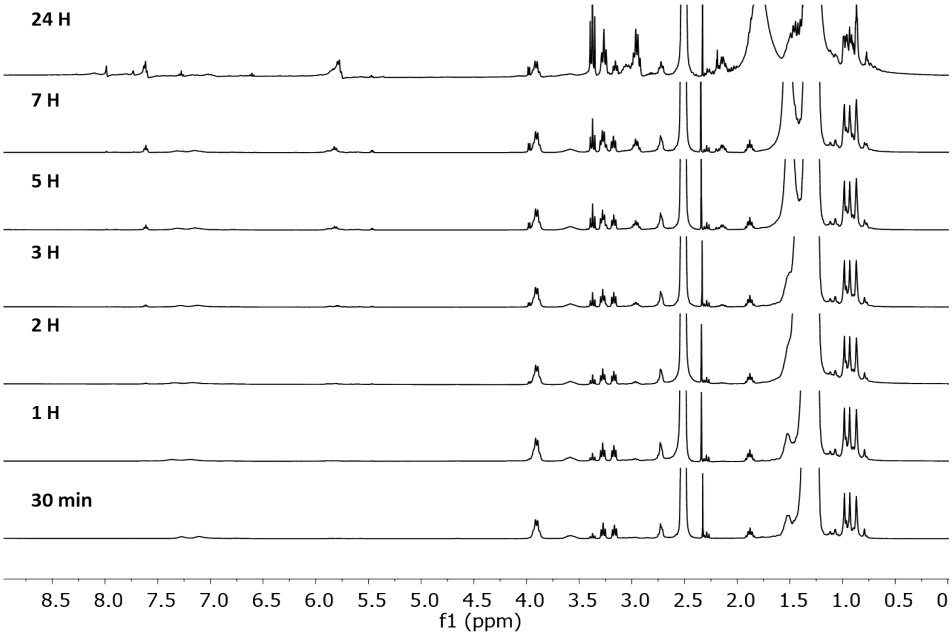

Figure S18. Stacked ^{1}H NMR spectra for the kinetics of IPDI-PU depolymerization with hexamethylenediamine as nucleophile with 0,3 eq. of TBD:MSA as catalyst at 130 °C. (DMSO-d_6, 300 MHz, 298 K)

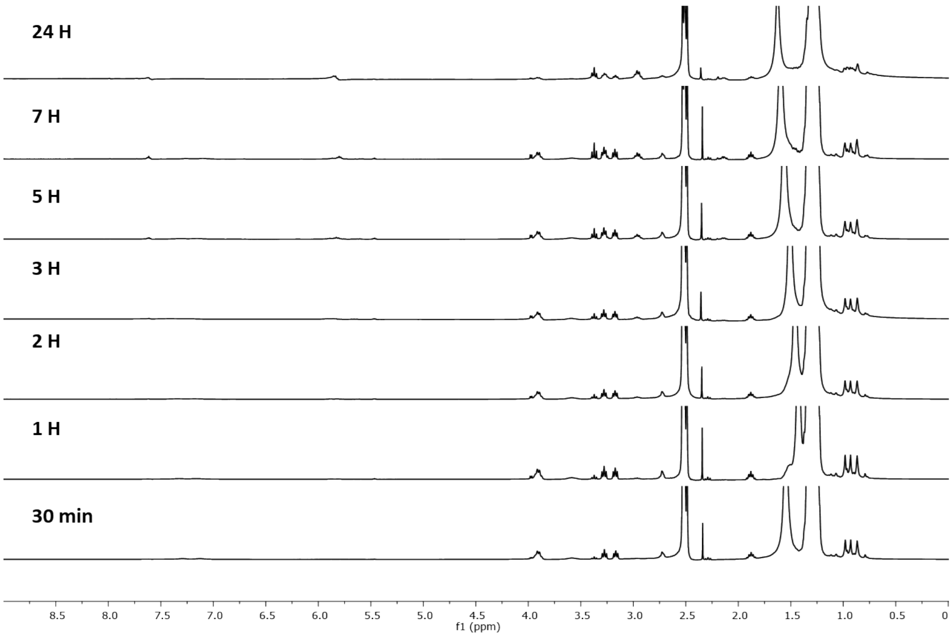

Figure S19. Stacked ^{1}H NMR spectra for the kinetics of IPDI-PU depolymerization with hexamethylenediamine as nucleophile with 0,45 eq. of TBD:MSA as catalyst at 130 °C. (DMSO-d_6, 300 MHz, 298 K)

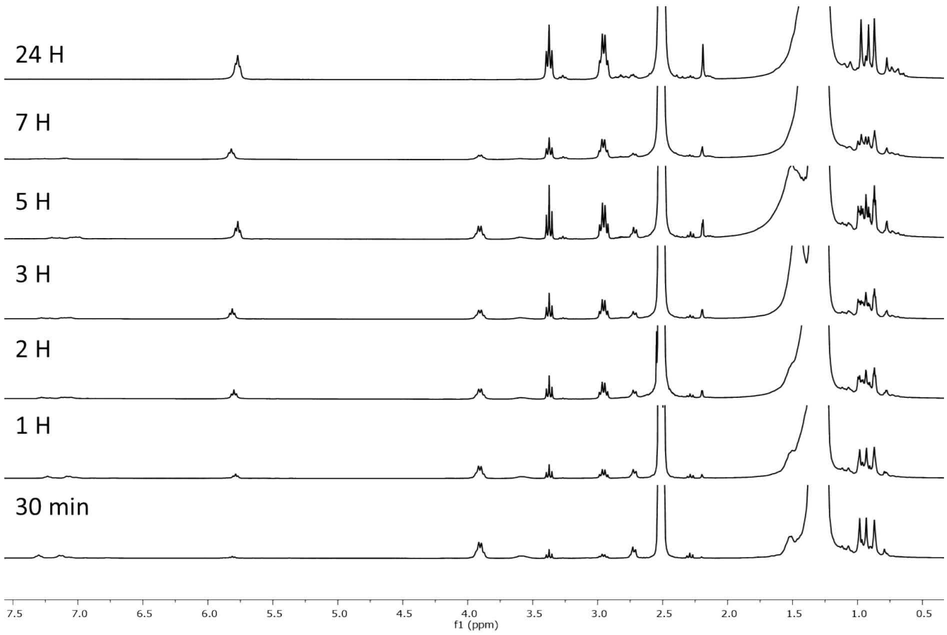

Figure S20. Stacked ^{1}H NMR spectra for the kinetics of IPDI-PU depolymerization with hexamethylenediamine as nucleophile without catalyst at 160 °C. (DMSO-d_6, 300 MHz, 298 K)

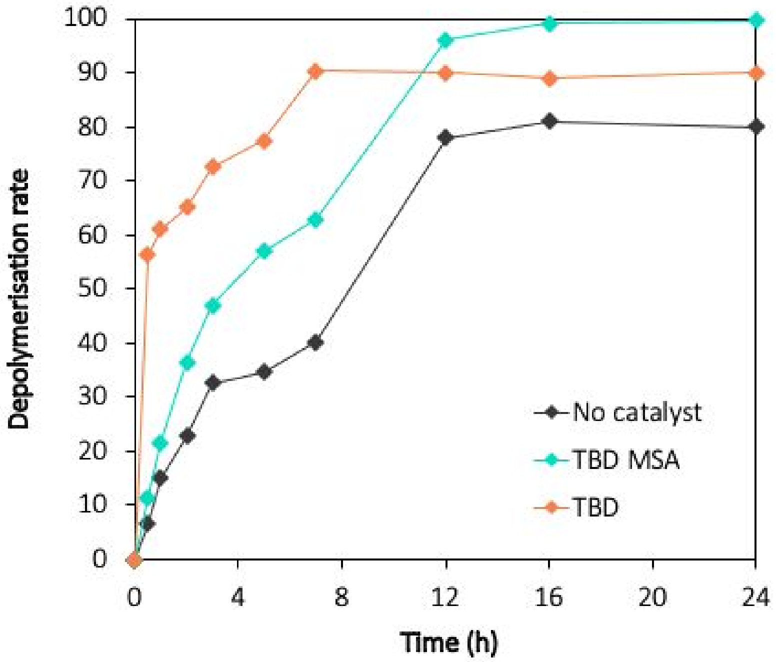

Figure S21. Kinetic plots for the depolymerization of IPDI-PU with hexamethylene diamine with different catalysts and without catalyst. *Reaction conditions: IPDI-PU (1 eq.), hexamethylene diamine (10 eq.), 24 h, 160* °C.

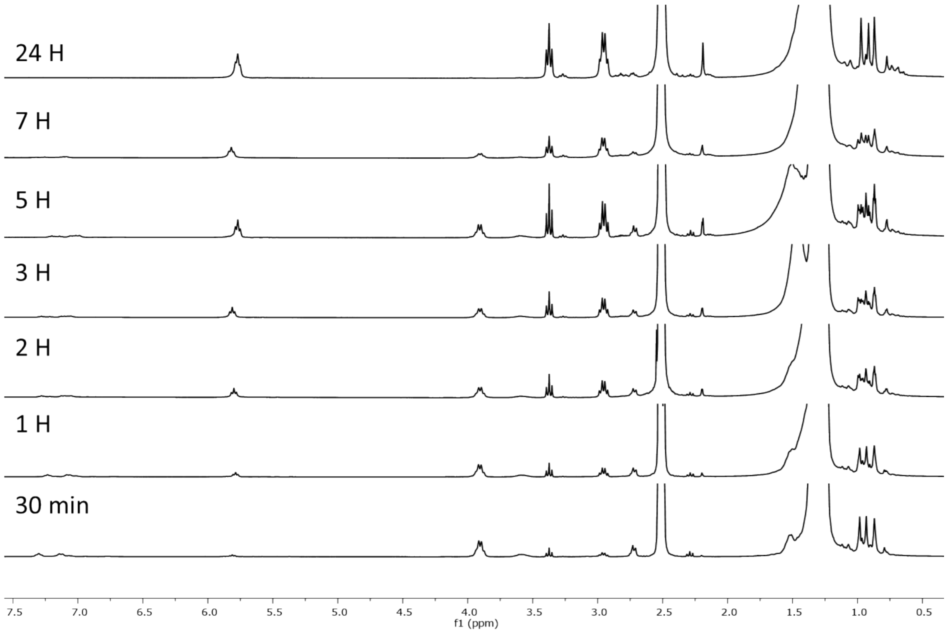

Figure S22. Stacked ^{1}H NMR spectra for the kinetics of IPDI-PU depolymerization with hexamethylenediamine as nucleophile without catalyst at 160 °C. (DMSO-d_6, 300 MHz, 298 K)

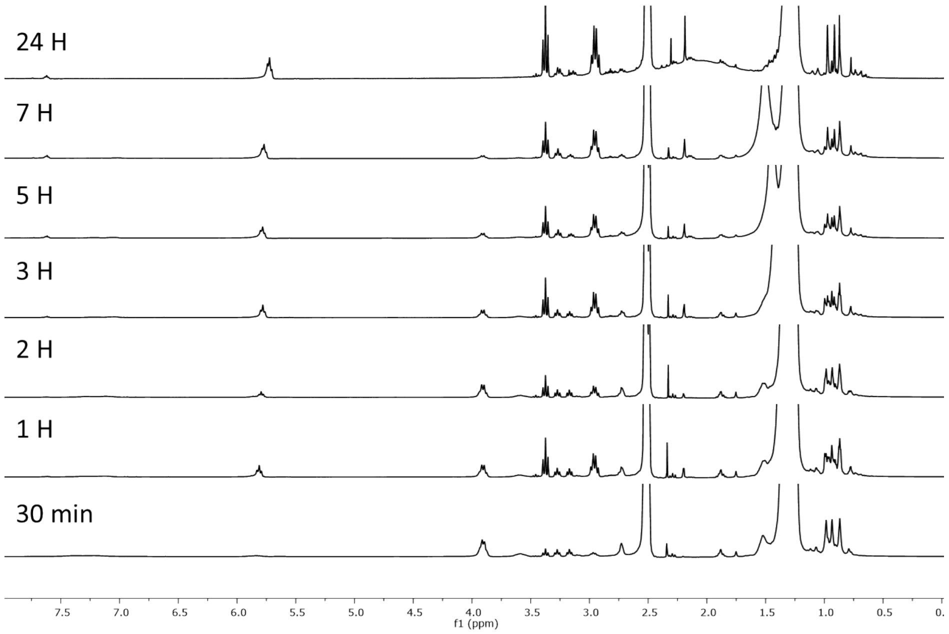

Figure S23. Stacked ^{1}H NMR spectra for the kinetics of IPDI-PU depolymerization with hexamethylenediamine as nucleophile with TBD as catalyst at 160 °C. (DMSO-d_6, 300 MHz, 298 K)

^{1}H NMR spectra of the crude products for the depolymerization of IPDI-PU with different amines

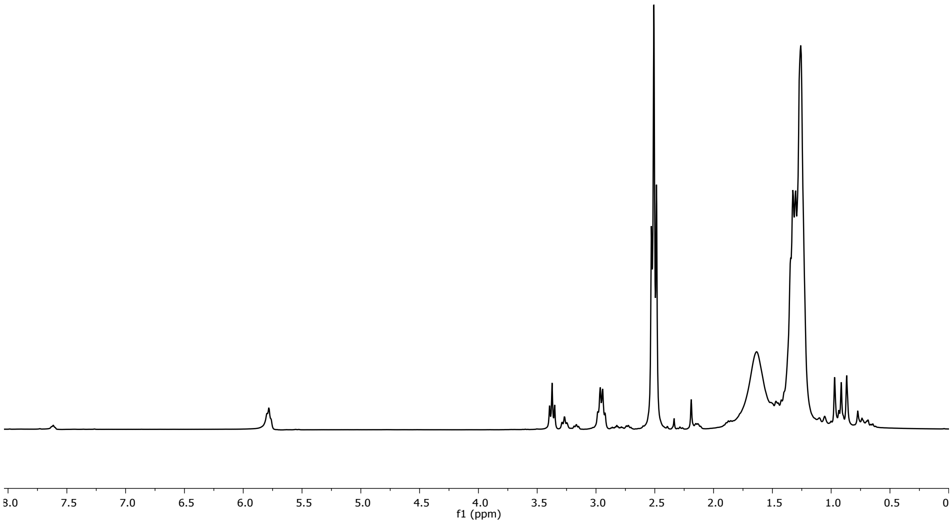

Figure S24. ^{1}H NMR spectra in DMSO-d_6 of the crude product resulting from the depolymerization of IPDI-PU with hexamethylene diamine as nucleophile. (DMSO-d_6, 300 MHz, 298 K)

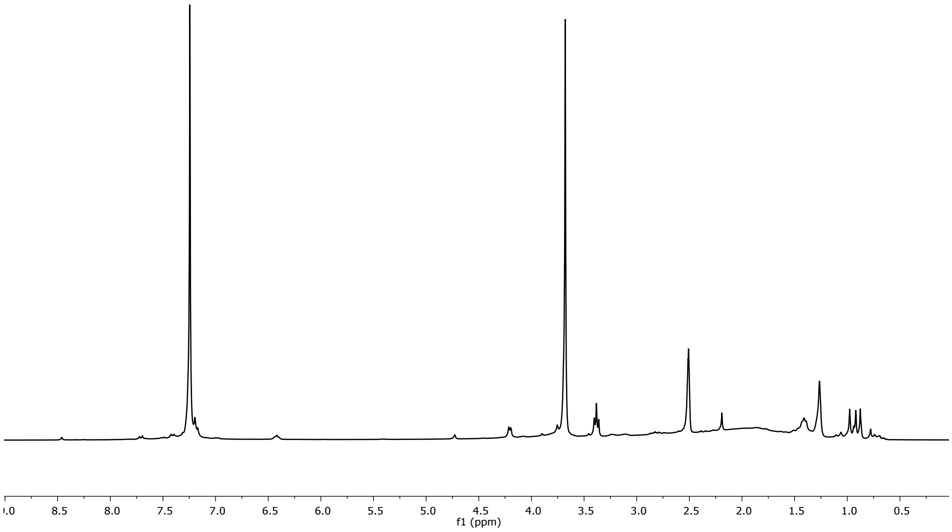

Figure S25. ^{1}H NMR spectra in DMSO-d_6 of the crude product resulting from the depolymerization of IPDI-PU with 1,4-phenylenedimethanamine as nucleophile. (DMSO-d_6, 300 MHz, 298 K)

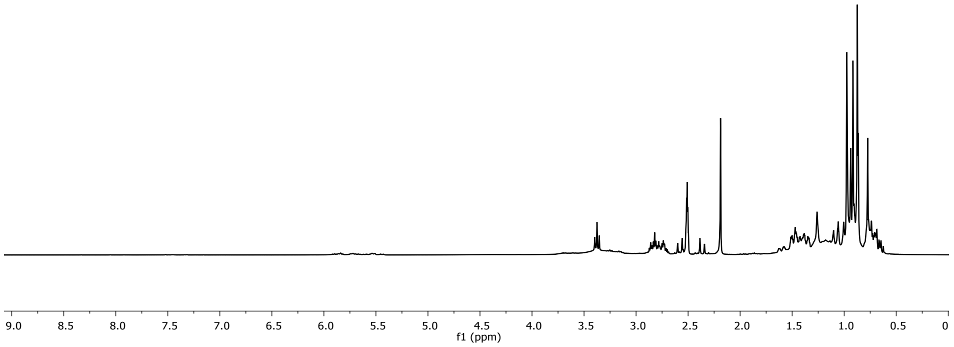

Figure S26. ^{1}H NMR spectra in DMSO-d_6 of the crude product resulting from the depolymerization of IPDI-PU with isophorone diamine as nucleophile. (DMSO-d_6, 300 MHz, 298 K)

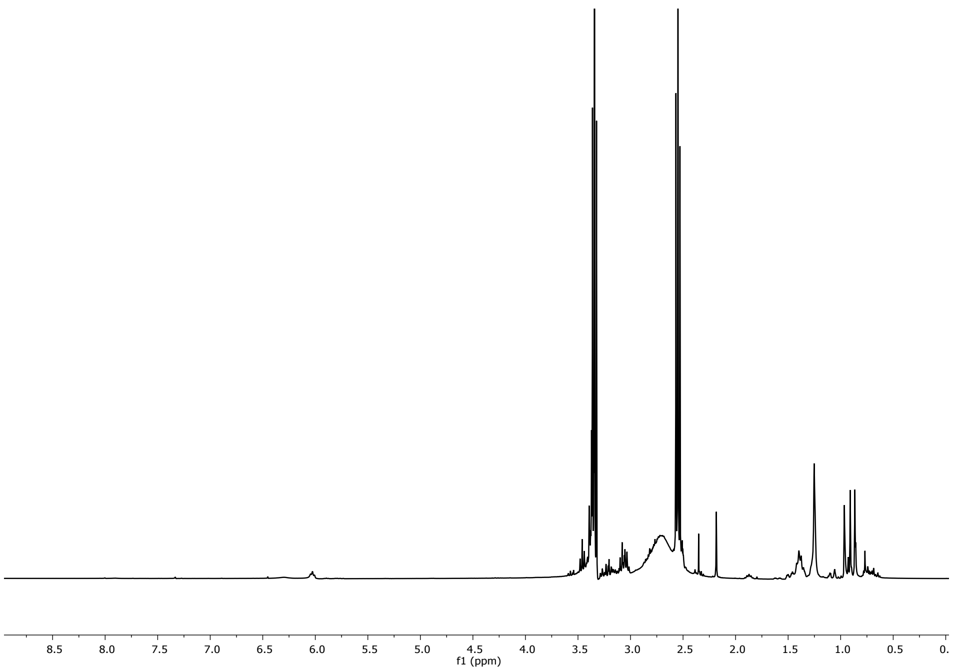

Figure S27. ^{1}H NMR spectra in DMSO-d_6 of the crude product resulting from the depolymerization of IPDI-PU with ethanolamine as nucleophile. (DMSO-d_6, 300 MHz, 298 K)

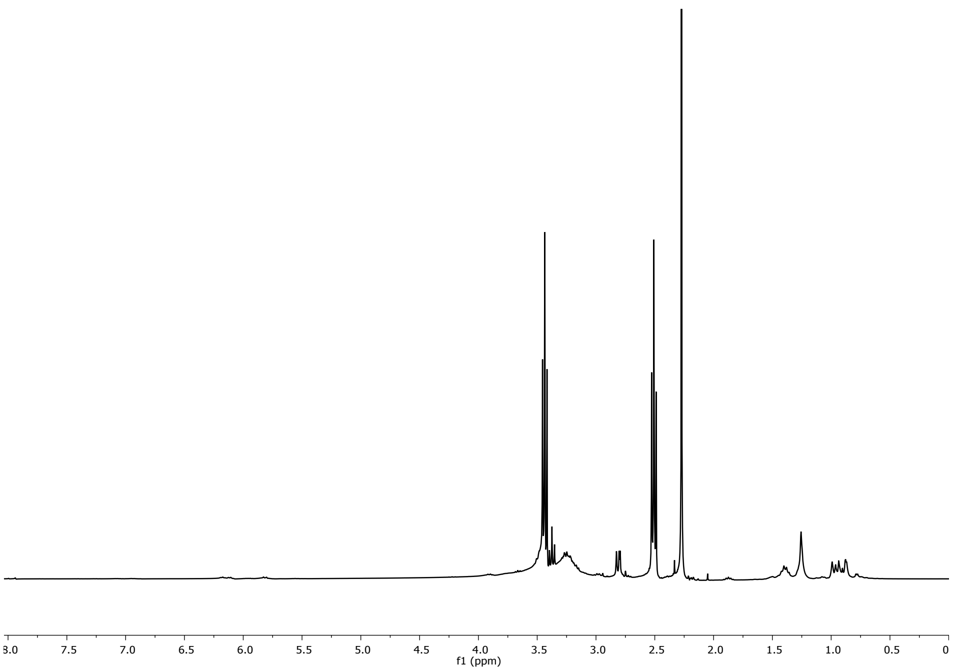

Figure S28. ^{1}H NMR spectra in DMSO-d_6 of the crude product resulting from the depolymerization of IPDI-PU with 2-(methylamino)ethan-1-ol as nucleophile. (DMSO-d_6, 300 MHz, 298 K)

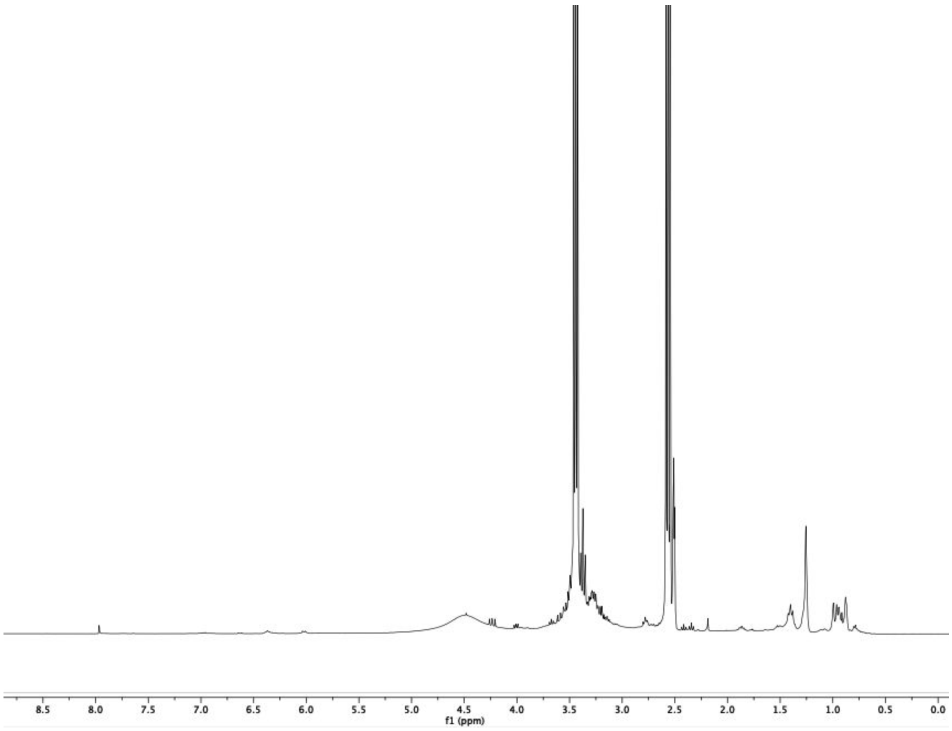

Figure S29. ^{1}H NMR spectra in DMSO-d_6 of the crude product resulting from the depolymerization of IPDI-PU with diethanolamine as nucleophile. (DMSO-d_6, 300 MHz, 298 K)

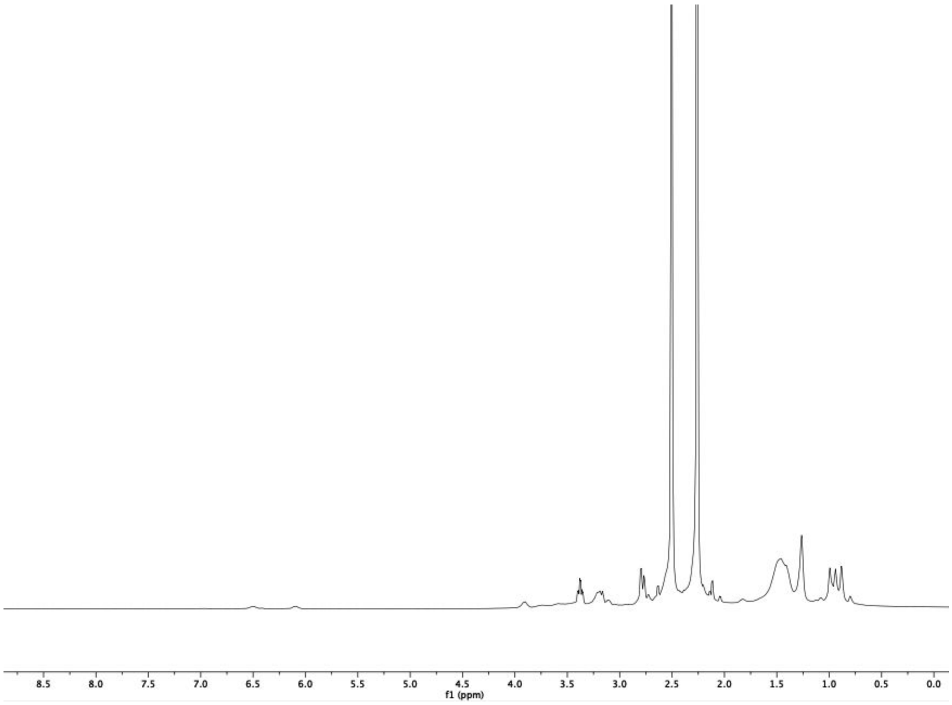

Figure S30. ^{1}H NMR spectra in DMSO-d_6 of the crude product resulting from the depolymerization of IPDI-PU with N,N,N-trimethylethane-1,2-diamine as nucleophile. (DMSO-d_6, 300 MHz, 298 K)

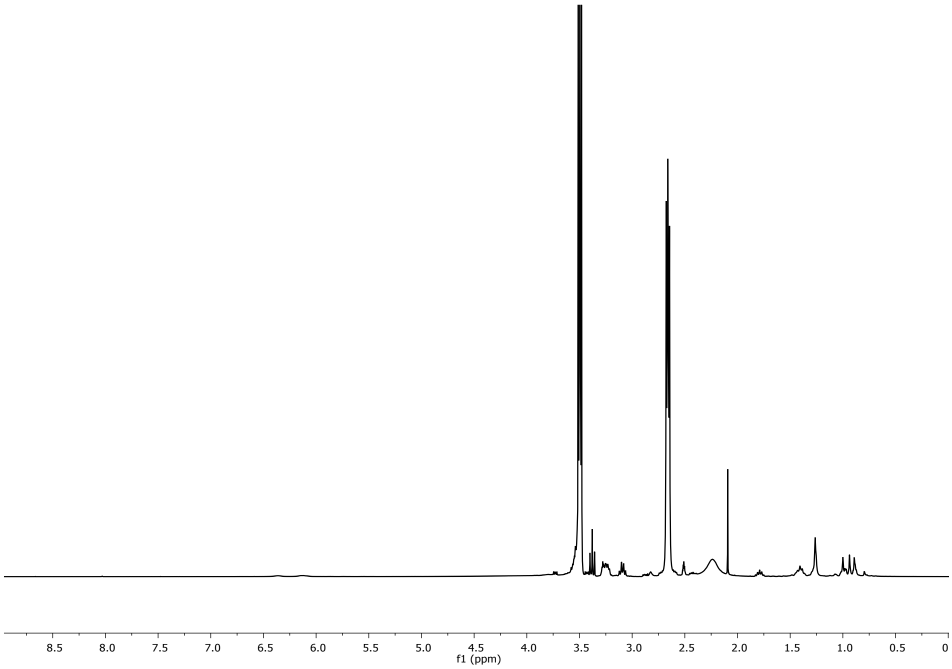

Figure S31. ^{1}H NMR spectra in DMSO-d_6 of the crude product resulting from the depolymerization of IPDI-PU with morpholine as nucleophile. (DMSO-d_6, 300 MHz, 298 K)

FTIR spectroscopy analysis for the reaction with methylamino)ethanol

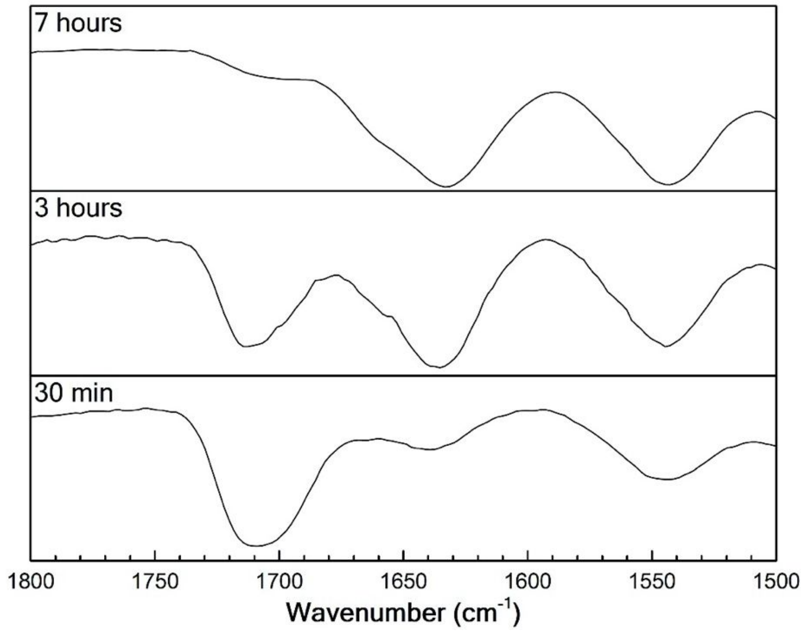

Figure S32. Kinetics for the the depolymerization of IPDI-PU with 2-(methylamino)ethan-1-ol as nucleophile.

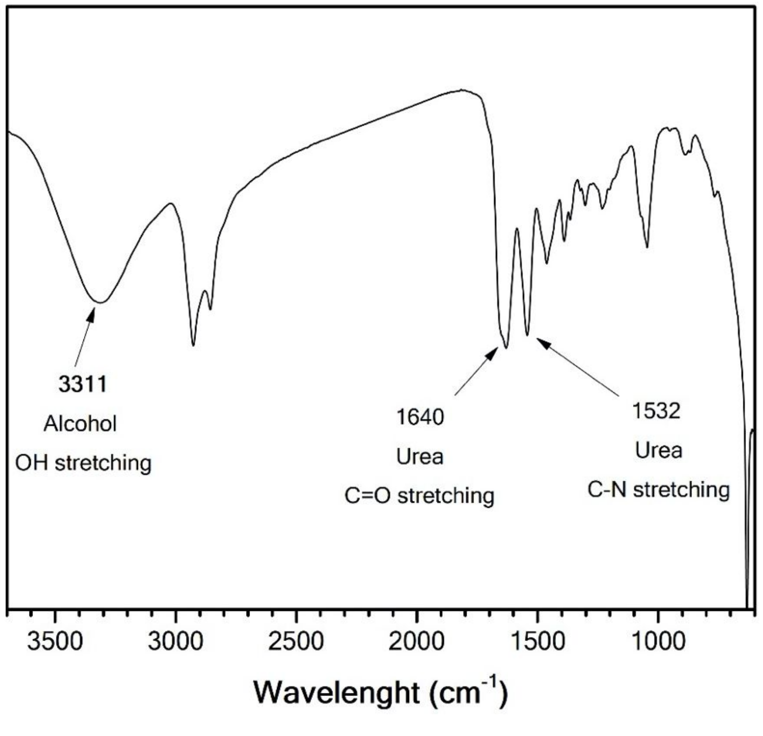

Figure S33. FTIR spectra of the crude product resulting from the depolymerization of IPDI-PU with 2-(methylamino)ethan-1-ol as nucleophile

HPLC-MS analysis for the reaction with 2-(methylamino)ethan-1-ol

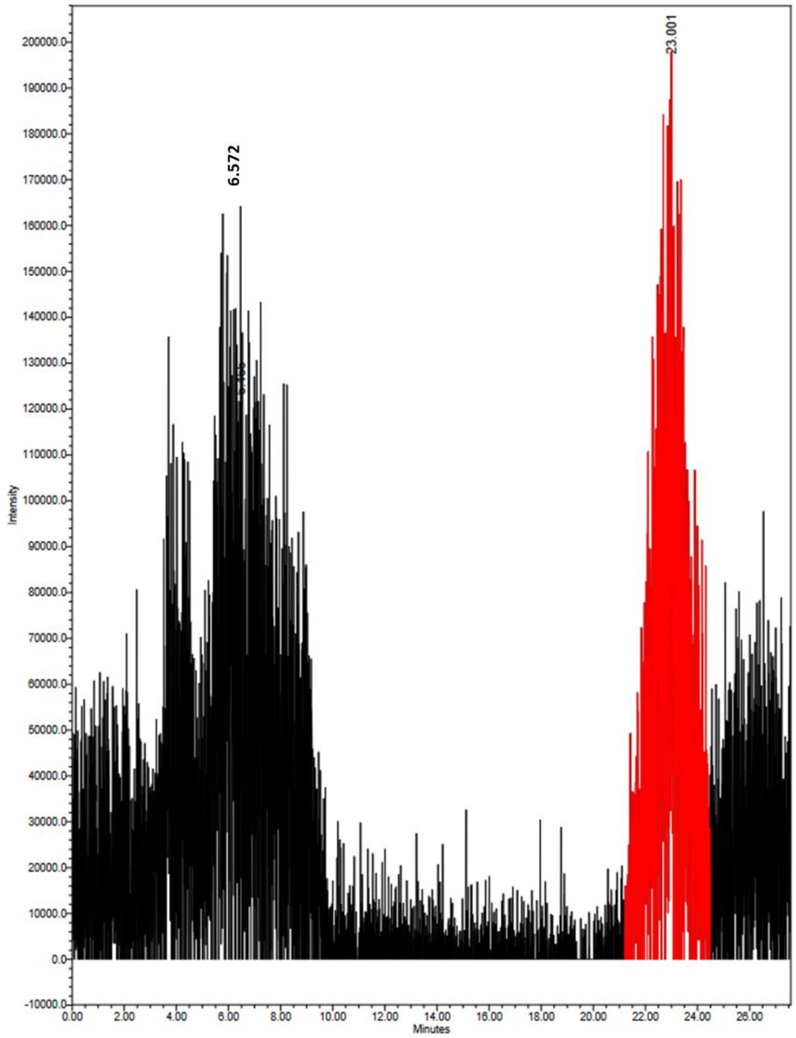

Figure S34. HPLC-MS complete chromatogram. Two main signals can be observed at 6.572 min and 23 min.

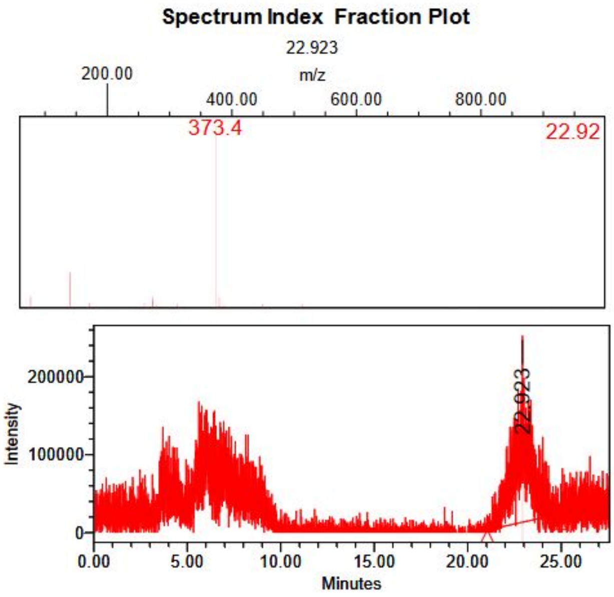

Figure S35. Spectrum index fraction plot and auto-scaled chromatogram showing the expected di-urea product (373.4 m/z) on the crude product of the reaction at 22.923 min retention time.

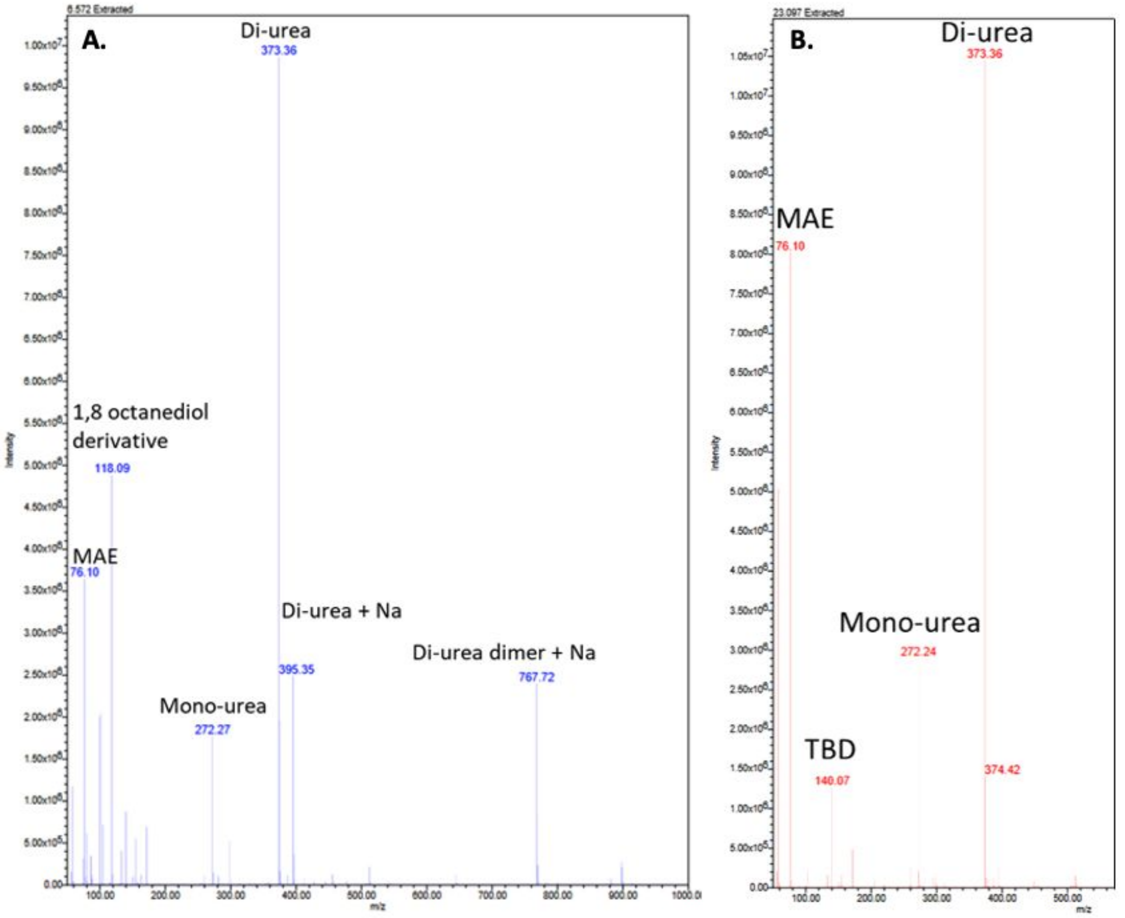

Figure S36. Mass spectra **A.** at 6.572 min retention time and **B.** at 23.007 min of retention time.

A. In this chromatogram, the signal characteristic of the 2-(methylamino)ethanol (MAE)can be observed at 76.10 m/z, the di-urea at 373.36 m/z, the di-urea with sodium at 395.35 m/z and the corresponding dimer with sodium at 767.72 m/z, are clearly observed. Different signals from

the fragmentation of some of the products are also observed. A signal at 272.27 m/z is identified as corresponding to the mono-urea which was confirmed to appear as a consequence of fragmentation during the analysis since this signal is also present in the spectrum of the lone di-urea. A signal at 118.09 m/z corresponds to the fragmentation of 1,8-octanediol which can be observed in the spectrum of the crude product of the reaction and the spectrum of the commercial product. Interestingly, the expected signal at 146 m/z does not appear, neither for the crude product nor for the commercial product. It can be because of the harsh ionization conditions applied on the mass spectrometer, the 1,8-octanediol could be fragmented to give heptanol.

B. The characteristic signal of 2-(methylamino)ethanol (MAE) (76.10 m/z) can be identified as well as the signal for TBD (at 140.07 m/z) and for the di-urea product (373.36 m/z) and the corresponding fragmentation into mono-urea (272.24 m/z).

The chromatogram of the lone IPDA presents two characteristic signals (at 170 m/z and at 340 m/z, corresponding to the dimer) which cannot be identified in none of these chromatograms of the crude product of the reaction, which clearly atests that no IPDA was obtained from the depolymerization IPDI-PU with 2-(methylamino)ethanol.

Table S1. Different products identified in the crude product by HPLC-MS and their corresponding m/z.

Molecule	Observed?	Fragmentation product	m/z
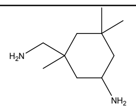 IPDA	No	N.A	170.18
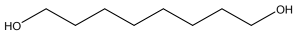 1,8-octanediol	Yes (only fragmentation product)	Heptanol	146 (1,8-octanediol) heptanol (116.09)
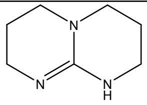 TBD	Yes	N.A	140.01
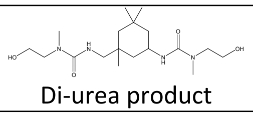 Di-urea product	Yes	Mono-urea	373.36
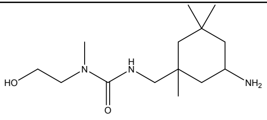 Mono-urea product	Yes	Product of the fragmentation of the di-urea	272.24

^{1}H NMR spectra of the crude products for the depolymerizations of TDI-PU

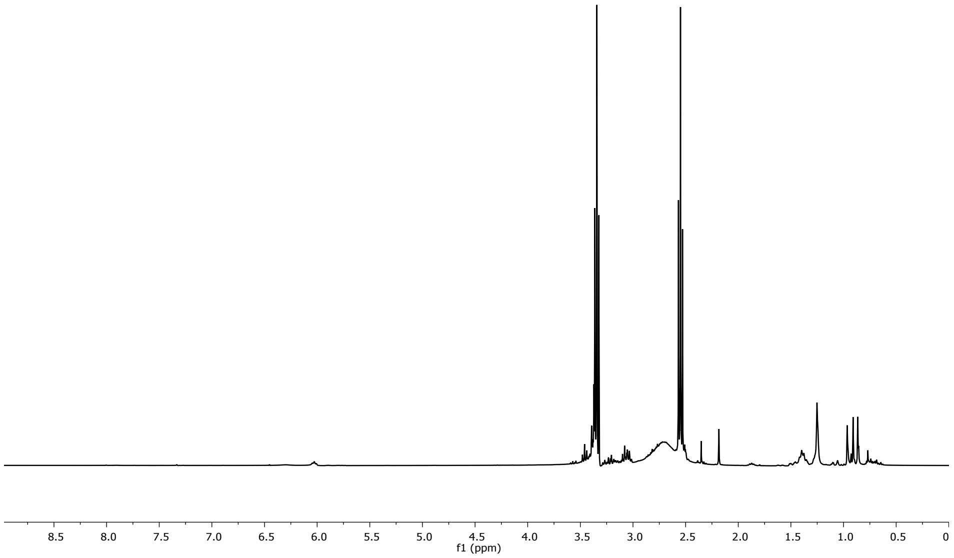

Figure S37. ^{1}H NMR spectra in DMSO-d_6 of the crude product resulting from the depolymerization of IPDI-PU with ethanolamine as nucleophile. (DMSO-d_6, 300 MHz, 298 K)

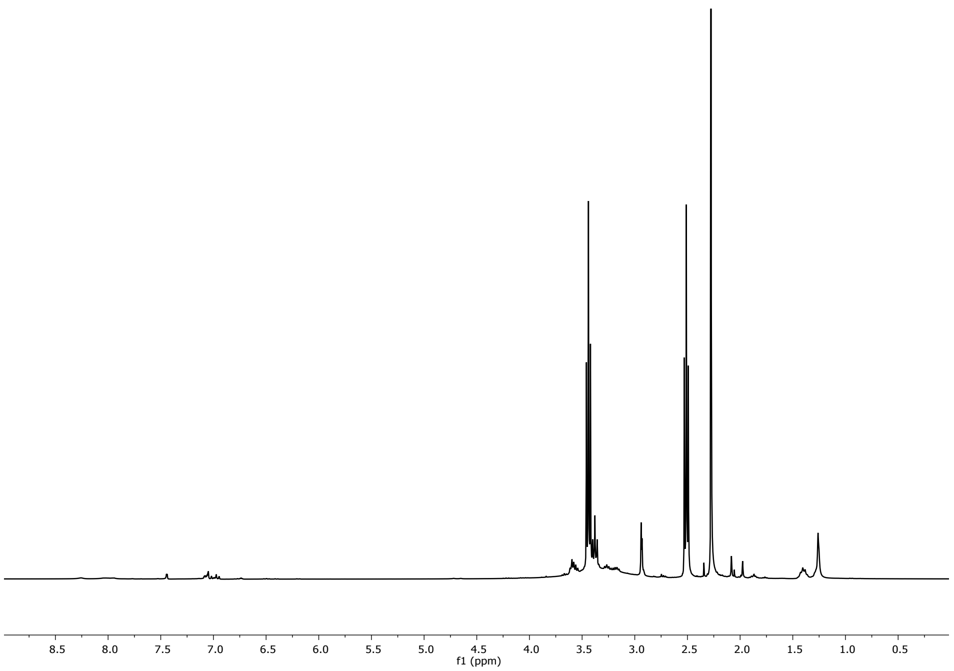

Figure S38. ^{1}H NMR spectra in DMSO-d_6 of the crude product resulting from the depolymerization of TDI-PU with 2-(methylamino)ethan-1-ol as nucleophile. (DMSO-d_6, 300 MHz, 298 K)

Comparison between depolymerization of IPDI-PU with secondary and primary amines

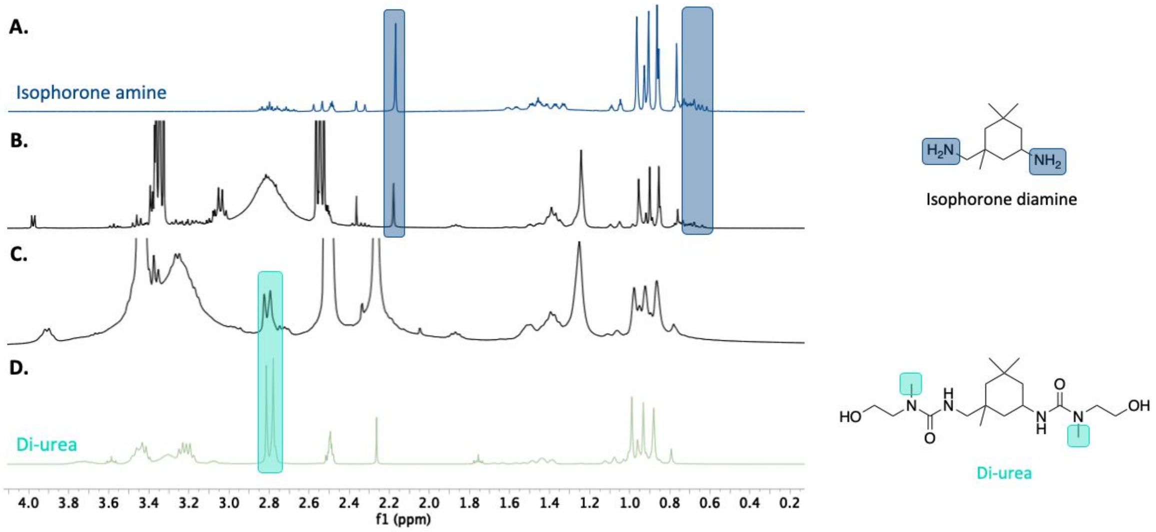

Figure S39. Selective carbamate breakage by secondary amines. **A.** ^{1}H NMR spectrum of isophorone diamine, **B.** crude product of the non-selective depolymerization of IPDI-PU with ethanolamine as nucleophile, **C.** crude product of the selective depolymerization of IPDI-PU with 2-(methylamino)ethan-1-ol as nucleophile and **D.** ^{1}H NMR spectrum of di-urea. (DMSO-d_6, 300 MHz, 298 K)

^1H NMR spectra of the crude products for the depolymerization of IPDI-PU with 2-(methylamino)ethan-1-ol with different catalysts and various temperatures

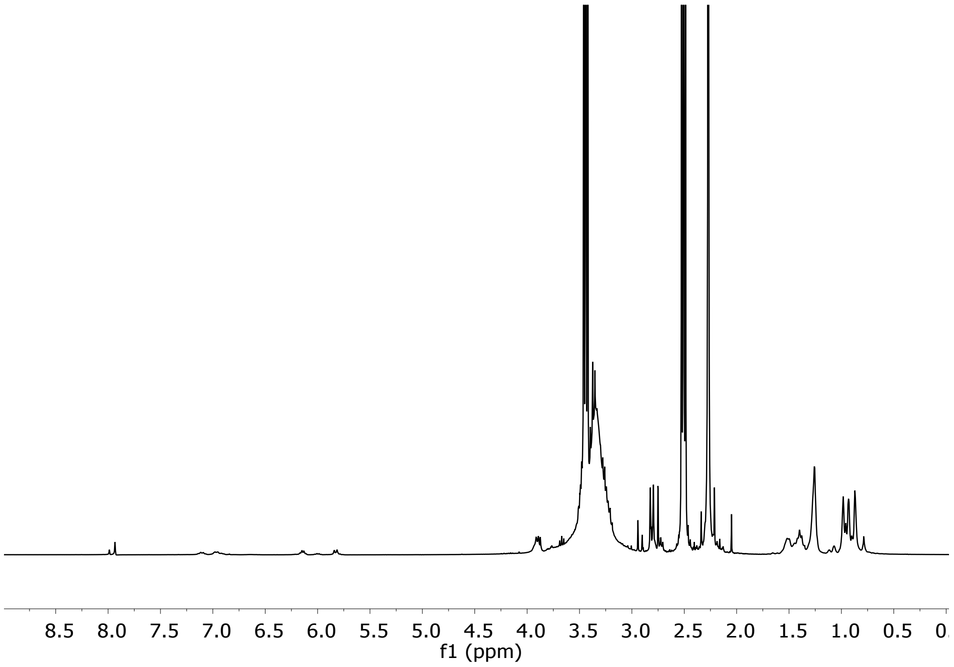

Figure S40. ^1H NMR spectra in DMSO-d_6 of the crude product resulting from the depolymerization of IPDI-PU with 2-(methylamino)ethan-1-ol as nucleophile without catalyst. (DMSO-d_6, 300 MHz, 298 K)

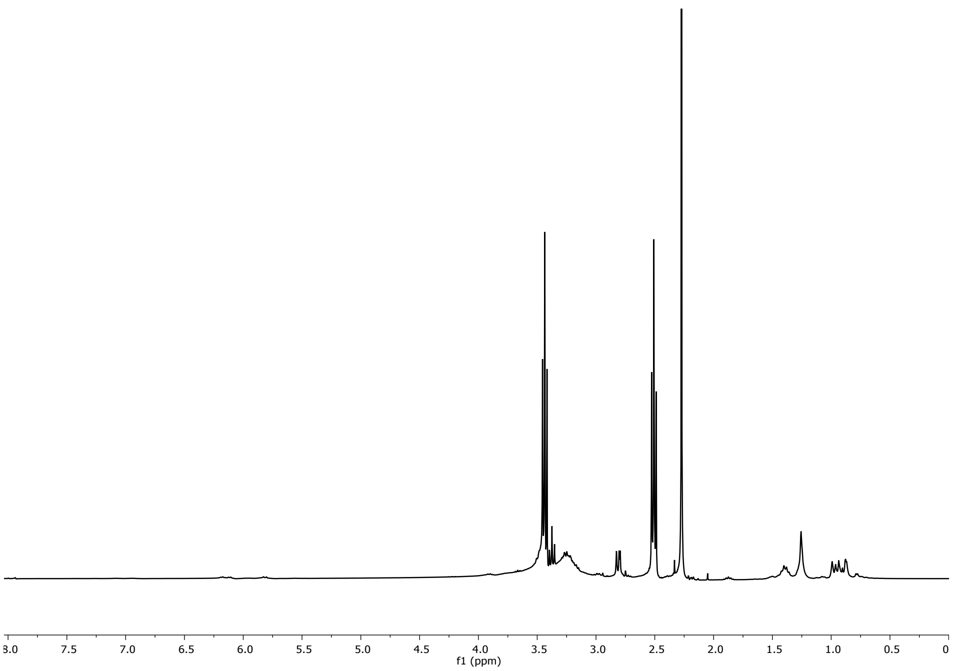

Figure S41. ^{1}H NMR spectra in DMSO-d_6 of the crude product resulting from the depolymerization of IPDI-PU with 2-(methylamino)ethan-1-ol as nucleophile with TBD:MSA. (DMSO-d_6, 300 MHz, 298 K)

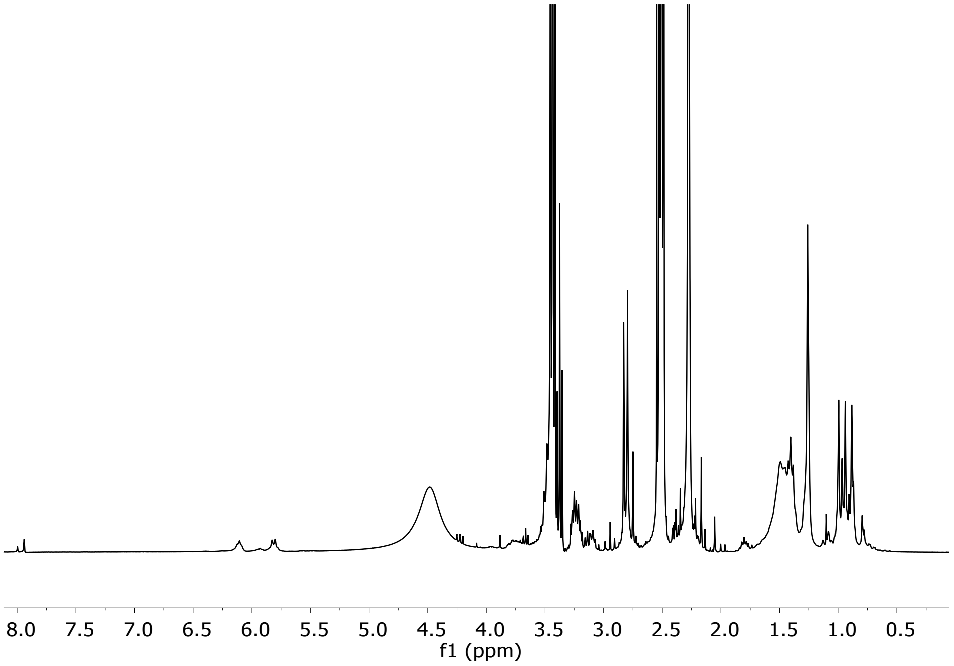

Figure S42. ^{1}H NMR spectra in DMSO-d_6 of the crude product resulting from the depolymerization of IPDI-PU with 2-(methylamino)ethan-1-ol as nucleophile with TBD. (DMSO-d_6, 300 MHz, 298 K)

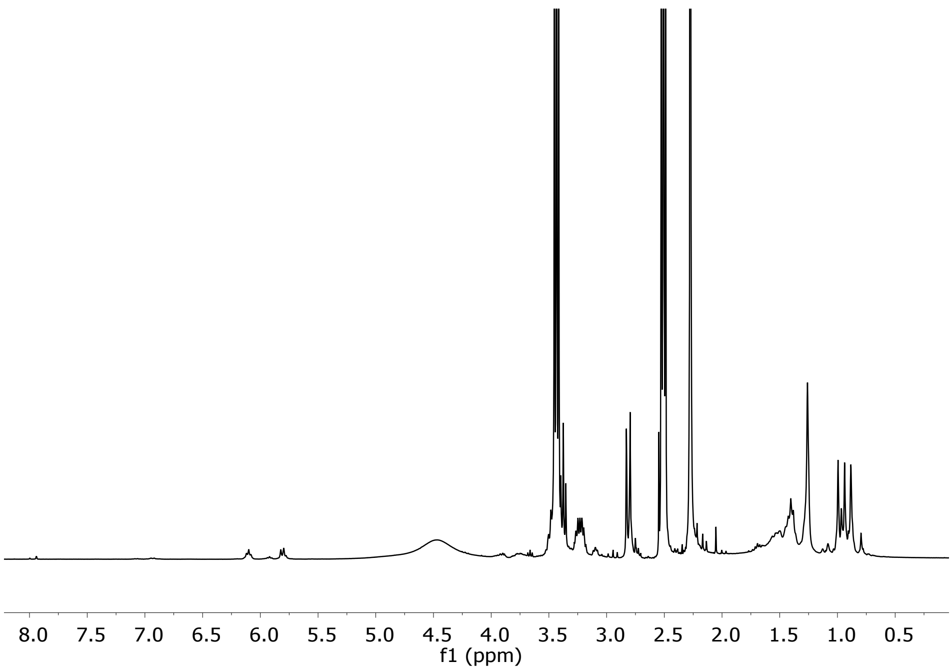

Figure S43. ^{1}H NMR spectra in DMSO-d_6 of the crude product resulting from the depolymerization of IPDI-PU with 2-(methylamino)ethan-1-ol as nucleophile with DBU. (DMSO-d_6, 300 MHz, 298 K)

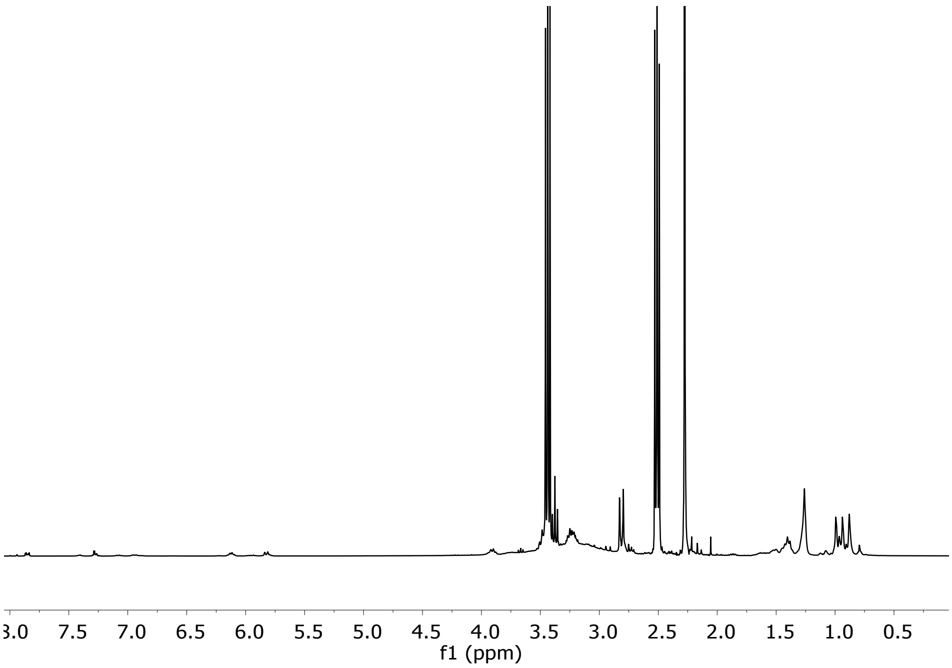

Figure S44. ^{1}H NMR spectra in DMSO-d_6 of the crude product resulting from the depolymerization of IPDI-PU with 2-(methylamino)ethan-1-ol as nucleophile with DBU:BA. (DMSO-d_6, 300 MHz, 298 K)

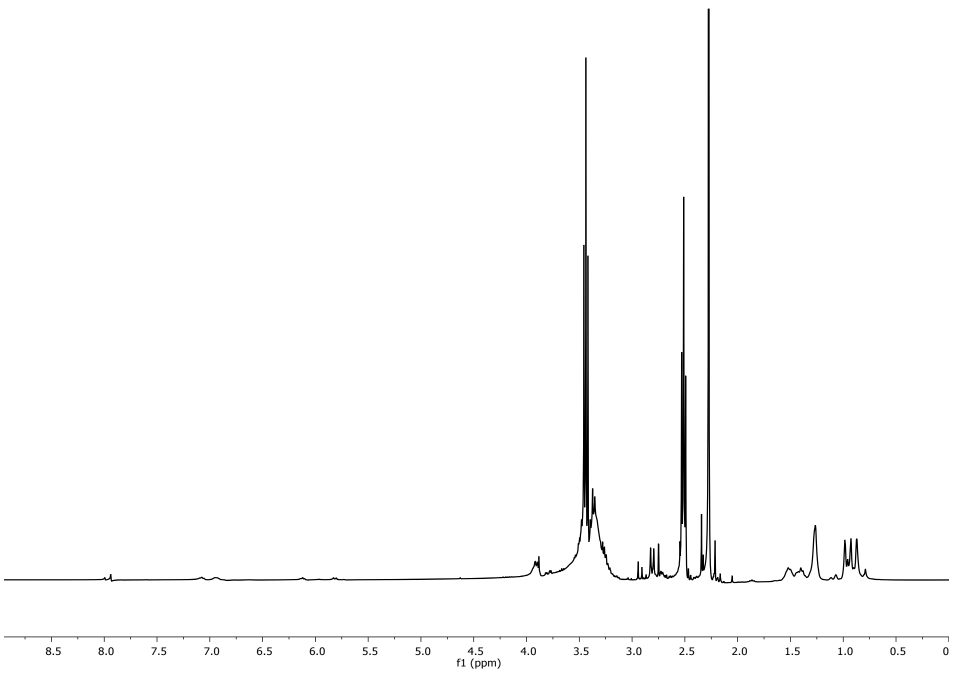

Figure S45. ^1H NMR spectra in DMSO-d_6 of the crude product resulting from the depolymerization of IPDI-PU with 2-(methylamino)ethan-1-ol as nucleophile at 130 °C. (DMSO-d_6, 300 MHz, 298 K)

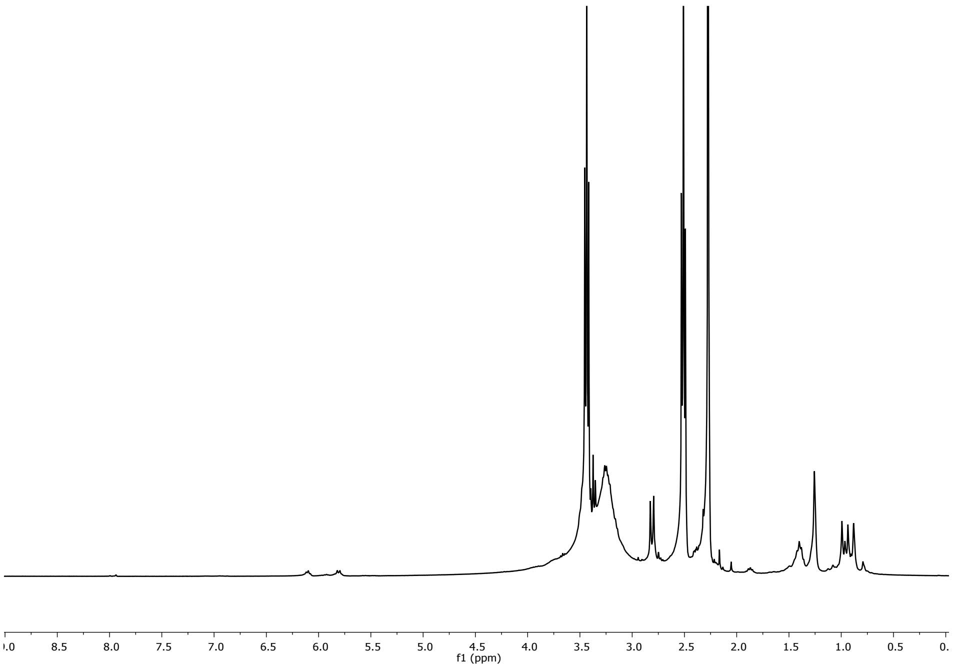

Figure S46. ^1H NMR spectra in DMSO-d_6 of the crude product resulting from the depolymerization of IPDI-PU with 2-(methylamino)ethan-1-ol as nucleophile at 190 °C. (DMSO-d_6, 300 MHz, 298 K)

MALDI analysis of the polyol

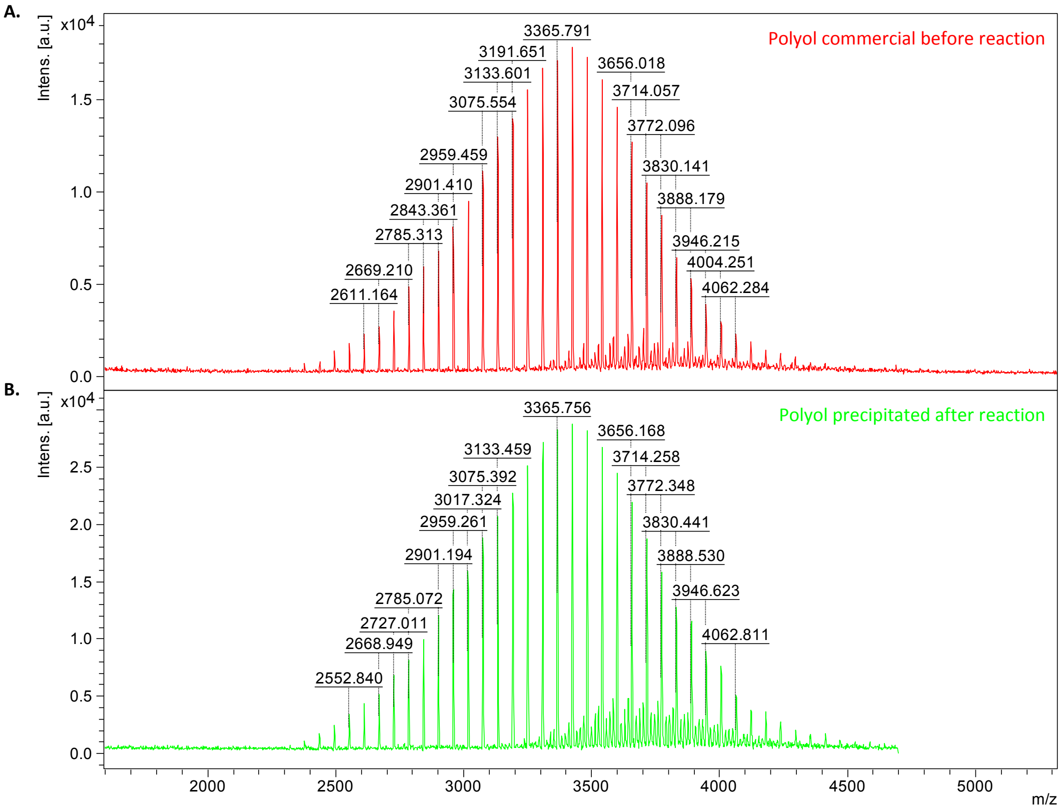

Figure S47. MALDI analysis of the polyol A. before the polymerisation and **B.** the recovered polyol after depolymerisation and purification.

^{1}H NMR spectra of the crude products for the depolymerizations of the commercial foams

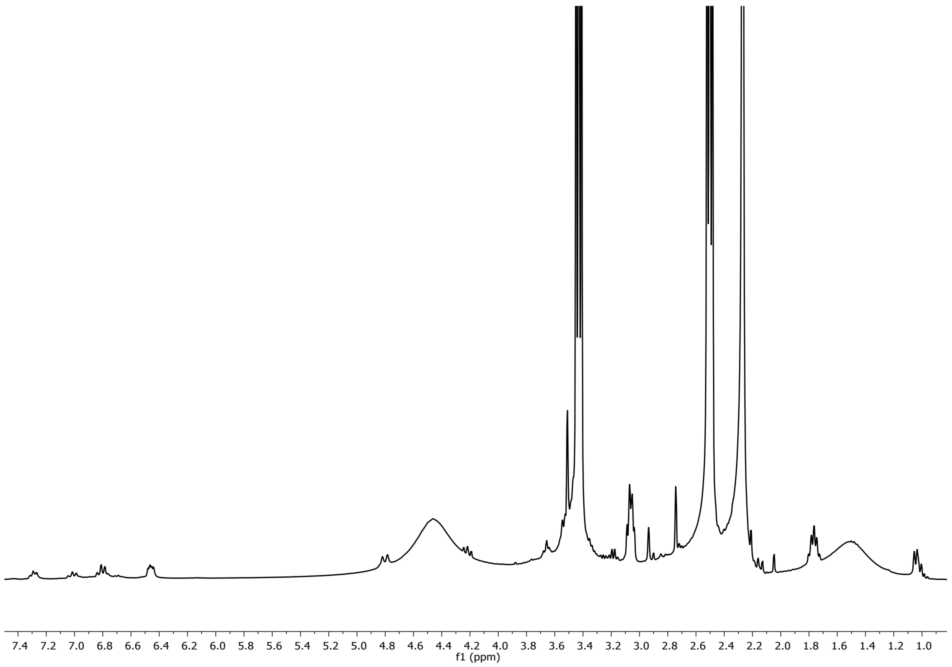

Figure S48. ^{1}H NMR spectrum of depolymerised CPU-F2 crude product. The di-urea characteristic signal can be identified at δ = 2.94 ppm. (DMSO-d_6, 300 MHz, 298 K)

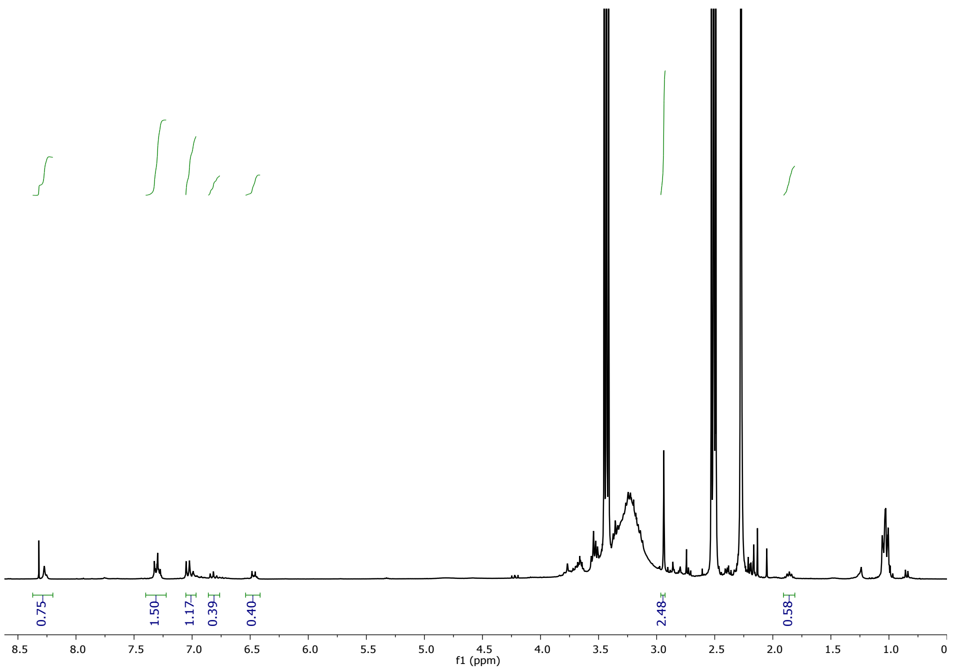

Figure S49. ^1H NMR spectrum of depolymerised CPU-F3 crude product. The di-urea characteristic signal can be identified at δ = 2.94 ppm. (DMSO-d_6, 300 MHz, 298 K)

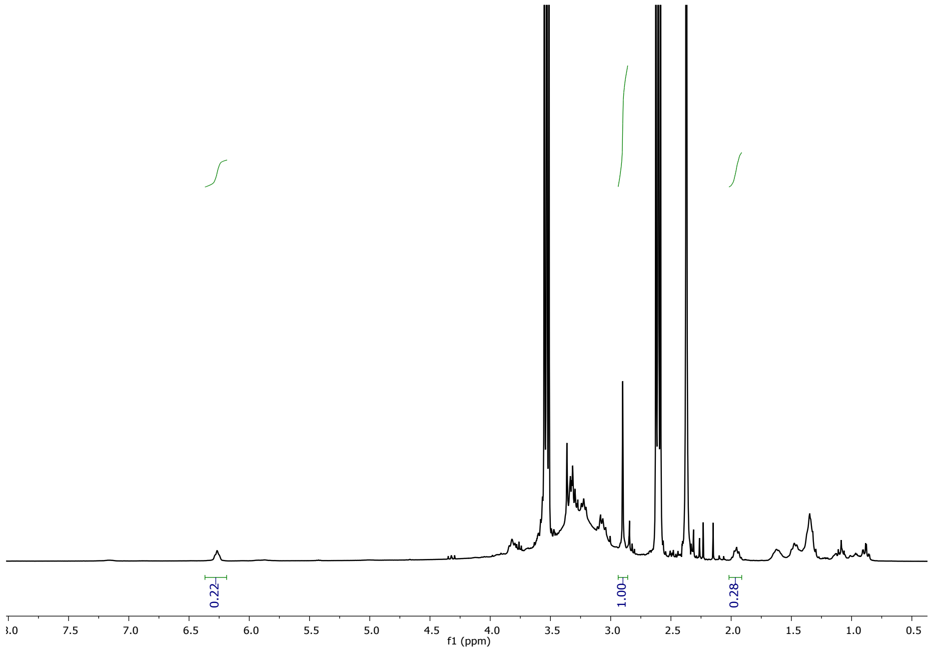

Figure S50. ^1H NMR spectrum of depolymerised CPU-F4 crude product. The di-urea characteristic signal can be identified at δ = 2.94 ppm. (DMSO-d_6, 300 MHz, 298 K)
